# Multicentre Double-Blind Placebo-Controlled Food Challenge Study in Children Sensitised to Cashew Nut

**DOI:** 10.1371/journal.pone.0151055

**Published:** 2016-03-11

**Authors:** Johanna P. M. van der Valk, Roy Gerth van Wijk, Anthony E. J. Dubois, Hans de Groot, Marit Reitsma, Berber Vlieg-Boerstra, Huub F. J. Savelkoul, Harry J. Wichers, Nicolette W. de Jong

**Affiliations:** 1 Department Of Internal Medicine, Allergology, Erasmus MC, Rotterdam, the Netherlands; 2 Department of Pediatric Pulmonology and Pediatric Allergology, University Medical Centre Groningen, GRIAC Research Institute, University of Groningen, Groningen, the Netherlands; 3 Department of Pediatric Allergology, Diaconessenhuis Voorburg, RdGG, Delft, the Netherlands; 4 Food and Biobased Research, Wageningen University and Research Centre, Wageningen, the Netherlands; 5 Emma Children’s Hospital, Pediatric Respiratory Medicine and Allergy, Academic Medical Centre, University of Amsterdam, Amsterdam, the Netherlands; 6 Laboratory of Cell Biology and Immunology, Wageningen University and Research Centre, Wageningen, the Netherlands; North Carolina State University, UNITED STATES

## Abstract

**Background:**

Few studies with a limited number of patients have provided indications that cashew-allergic patients may experience severe allergic reactions to minimal amounts of cashew nut. The objectives of this multicentre study were to assess the clinical relevance of cashew nut sensitisation, to study the clinical reaction patterns in double-blind placebo-controlled food challenge tests and to establish the amount of cashew nuts that can elicit an allergic reaction.

**Methods and Findings:**

A total of 179 children were included (median age 9.0 years; range 2–17 years) with cashew nut sensitisation and a clinical history of reactions to cashew nuts or unknown exposure. Sensitised children who could tolerate cashew nuts were excluded. The study included three clinical visits and a telephone consultation. During the first visit, the medical history was evaluated, physical examinations were conducted, blood samples were drawn and skin prick tests were performed. The children underwent a double-blind placebo-controlled food challenge test with cashew nut during the second and third visits. The study showed that 137 (76.5%) of the sensitised children suspected of allergy to cashew nut had a positive double-blind placebo-controlled food challenge test, with 46% (63) manifesting subjective symptoms to the lowest dose of 1 mg cashew nut protein and 11% (15) developing objective symptoms to the lowest dose. Children most frequently had gastro-intestinal symptoms, followed by oral allergy and skin symptoms. A total of 36% (49/137) of the children experienced an anaphylactic reaction and 6% (8/137) of the children were treated with epinephrine.

**Conclusion:**

This prospective study demonstrated a strikingly high percentage of clinical reactions to cashew nut in this third line population. Severe allergic reactions, including anaphylaxis requiring epinephrine, were observed. These reactions were to minimal amounts of cashew nut, demonstrated the high potency of this allergens.

**Trial Registration:**

www.ncbi.nlm.nih.gov/pubmed NTR3572

## Introduction

Only a limited number of clinical studies have been published on cashew nut allergy. Five relevant studies have been performed examining clinical symptoms [1]. All of these studies were based on a limited number of patients, varying between 16 and 47 participants. Cashew-allergic patients most frequently show skin symptoms, followed by respiratory and gastro-intestinal symptoms. Studies have shown that a small amount of cashew nut allergen may cause severe clinical reactions, suggesting a high potency of this nut, comparable to that of other tree nuts and peanuts [2]. The study by Davoren et al. reported that 30% of the peanut and 74% of the cashew nut sensitised patients with peanut and tree nut allergy developed an anaphylactic reaction after allergen ingestion. Moreover, in this study, 5 of 27 patients with cashew nut allergy experienced an allergic reaction after only skin or mucosal contact. One of these five patients developed anaphylaxis [2].

Clinical history, combined with the outcome of a skin prick test (SPT) and/or specific IgE (sIgE) test, is often used to establish the diagnosis of cashew nut allergy. The gold standard, however, is the double-blind placebo-controlled food challenge (DBPCFC) test.

The objectives of this study were to assess the clinical relevance of cashew nut sensitisation, to study the clinical reaction patterns and the severity of symptoms during the DBPCFC tests with cashew nut and to establish the amount of cashew nut that can elicit an allergic reaction.

## Material and Methods

### Study design and patient selection

**Fig 1:**
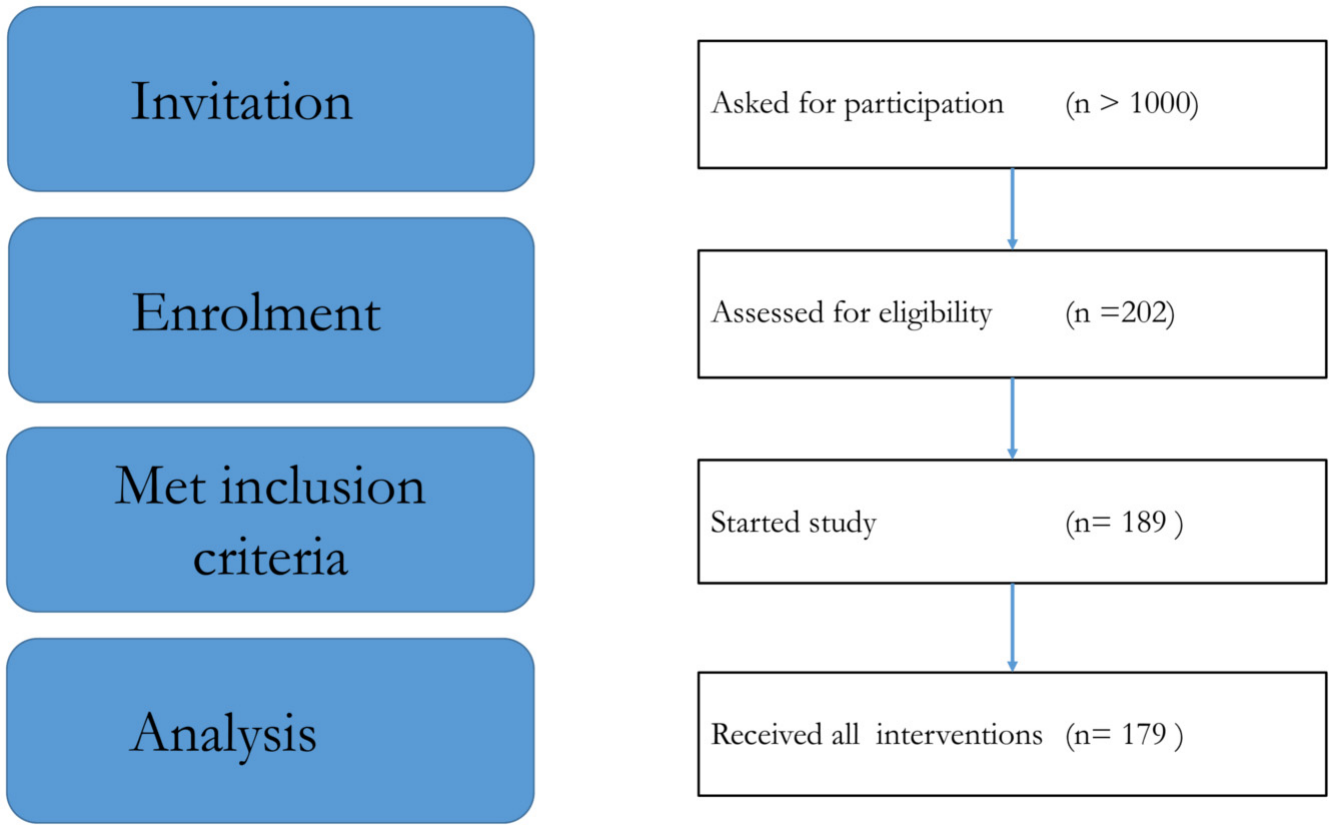
Flowchart of patient inclusion.

This study was a collaboration of three tertiary care centres for food allergy and the Research Centre Wageningen, the Netherlands. Consecutive new children and children known to have a sensitisation to cashew nut (sIgE and/or SPT) and a history of previous reaction(s) to cashew nut or unknown exposure were asked to participate in this study. More than 1000 children from this tertiary care population between 2 and 17 years of age were asked to participate. Approximately 1 in 3 parents of children who responded to the invitation (40%), agreed. Children with high sIgE (≥ 100 kU/l) to cashew nut and/or anaphylactic reactions after cashew nut ingestion in the past were also included. Sensitised children who could tolerate cashew nuts were excluded. All children were included in the study between May 2012 and March 2015. The last enrolled child finished the study in May 2015. The inclusion and exclusion criteria of the study are shown in Table 1 and a flowchart of the patient inclusion is shown in Fig 1.

**Table 1:** Inclusion and exclusion criteria.

**Inclusion criteria**
Age between 2 and 17 years.
Positive skin prick test (mean wheal diameter ≥ 3 mm Ø and HEP-index area ≥ 0.4 and/ or detectible sIgE (> 0.35 kU/L) to cashew nut.
History of previous positive reaction to cashew nut or unknown exposure.
Written informed consent from parents (2–12 years old), or parents and child (≥ 12 years old).
**Exclusion criteria**
History of severe or uncontrolled asthma (according to the physician’s assessment).
Autoimmune diseases, cardiovascular diseases or cancers.
Severe psychosocial problems.
The patient is allergic to one or more of the ingredients of the test food, unless a suitable substitute for the ingredient in question can be found.
Unable to stop taking antihistamine medication for a short period.
Use of beta-blockers.
Unable to speak and understand the Dutch language.

### Study procedure

The study program consisted of three clinical visits and a telephone consultation, as shown in Table 2. During the first visit, written informed consent was obtained (from parents of children (2–12 years old) and from parents and children (≥ 12 years old) and two medical history questionnaires were completed. Blood samples were drawn, SPT and physical examinations were conducted by a nurse and a physician. All children underwent a DBPCFC test with cashew nut in the second and third visits. The results of the challenge tests were discussed with the parents of the children by phone within a week after the DBPCFC test.

**Table 2:** Study program.

Visit 1	Visit 2	Visit 3	Telephone consultation	Final visit (optional)
Week 1	Week 4	Week 6	Week 7	Week 8
Written informed consent	DBPCFC test	DBPCFC test	Result DBPCFC test	Dietary advice
	Session 1	Session 2		
Medical history (2 questionnaires)				
Physical examination				
Blood samples (19 ml)				
Skin prick test				

Medical ethical approval for this study was obtained on 19 April 2012 and the study was registered in the Dutch trial register on 10 August 2012 (registered with administrative delay).

### Questionnaires

We used two questionnaires, which were specifically designed for this study. The medical history questionnaire contained 54 questions about general health, asthma and eczema. Also food allergies other than cashew nut allergy were evaluated by questions about the symptoms, the time between exposure and reaction, and the amount of allergenic food that caused the reaction. The dietary history questionnaire (12 questions) was used to identify allergic reactions in the past caused by cashew nut consumption. The type and severity of reaction was extensively evaluated and the amount of cashew nut causing the reaction was determined. The time between the reaction in history and the intake was also checked.

### Skin prick test

The children underwent a SPT with cashew nut, pistachio nut, hazelnut, peanut, mango and birch pollen extracts, a positive control (histamine 10 mg/ml ALK-Abello, Nieuwegein, the Netherlands) in duplicate and a negative control. All the extracts, except birch pollen (ALK 10.000 BU), were made according to a previously described method [3]. Cashew nuts (roasted, unsalted) and pistachio nuts, hazelnuts and peanuts (fresh, not roasted, unsalted nuts) were homogenised mechanically, ground with a mortar and pestle, defatted by ether extraction, and subsequently the extracts were air-dried. A 10% w/v extract in PBS (phosphate-buffered saline) with the pre-treated material was made and stored at -20°C in small aliquots. Before testing the aliquots were defrosted and mixed. Mango juice was prepared from small pieces of ripe mango fruit pulp, without skin or kernel.

SPTs were performed by applying a drop of the allergen extract on the skin of the volar part of the forearm. The extract was pierced through the skin barrier with a lancet. Twenty minutes after the skin tests, the contours of the wheal were encircled with a fine-tip pen and transferred to a record sheet by translucent tape. The area of the wheals was determined by using a scanner device (Hewlett Packard 2400c) in combination with software previously developed in our centre: Precise Automated Area Measurement of Skin Test (PAAMOST). The area of the allergen-induced wheal was divided by the mean area of the two positive histamine-induced wheal controls. This ratio was defined as the Histamine Equivalent Prick (HEP)-index area. The average wheal diameter was measured as well. An average wheal diameter ≥ 3 mm and a HEP-index area ≥ 0.4 was considered positive[4].

### In vitro tests

Serum samples were analysed for sIgE using the Siemens IMMULITE 2000 XPi Immunoassay System (Med. Imm. Laboratory; Reinier de Graaf Groep (RdGG). Levels above 0.35 kU/L were considered positive. sIgE against cashew nut, pistachio nut, hazelnut, peanut, mango and tree pollen were determined.

### Food challenge test

#### Procedure and recipe challenge test

Each patient underwent a DBPCFC test with cashew nut. The test food was administered in increasing amounts of 8 doses at time intervals of 30 minutes. Placebo and cashew nut challenges were randomly administered on separate days with at least a one-week interval. Validated and standardised food challenge material was used in the DBPCFC tests [5]. Roasted cashew nuts were provided by Intersnack, Doetinchem, the Netherlands. NIZO Food Research, Ede, the Netherlands prepared the low-fat food matrix (muffin dough). The food matrix predominantly consisted of wheat, sugar, gingerbread spice mix and coconut. The total volume/weight of the cashew nut gingerbread was 120 grams. The starting dose consisted of 1 mg cashew protein, followed by increasing doses of 3, 10, 30, 100, 300, 1000, 1736 mg cashew protein. Dose 8 consisted of the remainder of the 120 grams cashew nut gingerbread recipe. In children below the age of 4 years the challenge was stopped at step 7 (1000 mg cashew protein), because of the large amount of challenge material. The challenged doses are shown in Table 3.

**Table 3:** Challenge dosage DBPCFC test with cashew nut [5].

Dose steps	Cashew nut protein (mg)	Cashew nut protein cumulative (mg)	Cashew nut cumulative (number)*
1	1	1	0.01
2	3	4	0.03
3	10	14	0.10
4	30	44	0.30
5	100	144	1
6	300	444	3
7	1000	1444	10
8	1736	3180	22

* 1 cashew nut = approximately 700 milligrams.

#### Assessment and DBPCFC tests

The DBPCFC test was discontinued and considered positive when objective symptoms occurred, or when subjective symptoms re-occurred twice after the same dose of challenge material had been administered, three times consecutively[6], or when severe subjective symptoms persisted for more than one hour. If the child presented with the same symptoms on the placebo as on the verum day, the DBPCFC test was considered as undetermined. Anaphylaxis was defined as described in the EAACI Guidelines for Food Allergy and Anaphylaxis[7].

#### Procedure after the outcome of the DBPCFC test

In negative challenge test results, the child was advised to introduce cashew nuts at home. If the parents or child expected to experience problems with the introduction, a home introduction schedule developed by Vlieg-Boerstra et al.[8] and made available online [9] with increasing amounts of cashew nuts was recommended. These introduction schedules comprise instructions for parents and photographs with information on the required amounts of specific food for home introduction. The schedules were advised to improve the safety of the cashew nut introduction at home. Children with a positive DBPCFC test were advised to strictly avoid cashew nuts. If necessary, the participant was referred to a dietician after the DBPCFC test for extensive information and advice.

### Statistical analysis

In this descriptive study, the patient and the study characteristics were reported in median, ranges and proportions. All analyses were done using SPSS software, 20th edition.

## Results

### Study population

The study included a total of 179 children. The most commonly cited reason for not participating was that it was time consuming, burdensome for the child to undergo allergy testing (SPT and sIgE), and the fear of a reaction during the challenge test. The median age was 9.0 years (range 2–17 years), with 106 boys (59%) and 73 girls (41%). The children came from all over the Netherlands because the three participating medical research centres were spread across the country. All patients’ demographic and clinical characteristics are summarised in Table 4.

**Table 4:** Demographic and clinical characteristics.

**Total**	179	
**Gender**		
Male	106	(59%)
Female	73	(41%)
**Median age (years)**	9.0	(range 2–17)
**Atopic disease symptoms**		
Asthma	55	(31%)
Eczema	70	(39%)
Hay fever	94	(53%)
**Diagnostics**		
Median sIgE cashew nut (kU/l)	3.72	(range 0–100)
Median SPT (HEP-index area)	3.02	(range 0–15.16)
Diagnosis DBPCFC test		
Positive	137	(76.5%)
Negative	36	(20.1%)
Undecided	6	(3.4%)

### Questionnaires

Symptoms consistent with eczema were reported by 70 children (39%) and those for asthma by 55 children (31%). 94 children (53%) had symptoms consistent with hay fever. In 112 children (63%) consumption of, or contact with cashew nuts had elicited an allergic reaction before study entrance. These symptoms consisted mostly skin symptoms after cashew nut consumption in their history, followed by gastro-intestinal symptoms, respiratory symptoms, oral allergy symptoms and eye symptoms. The majority of the children reacted to cashew nuts as a single food ingested and not incorporated in other foods and to an amount of approximately one cashew nut.

**Fig 2:**
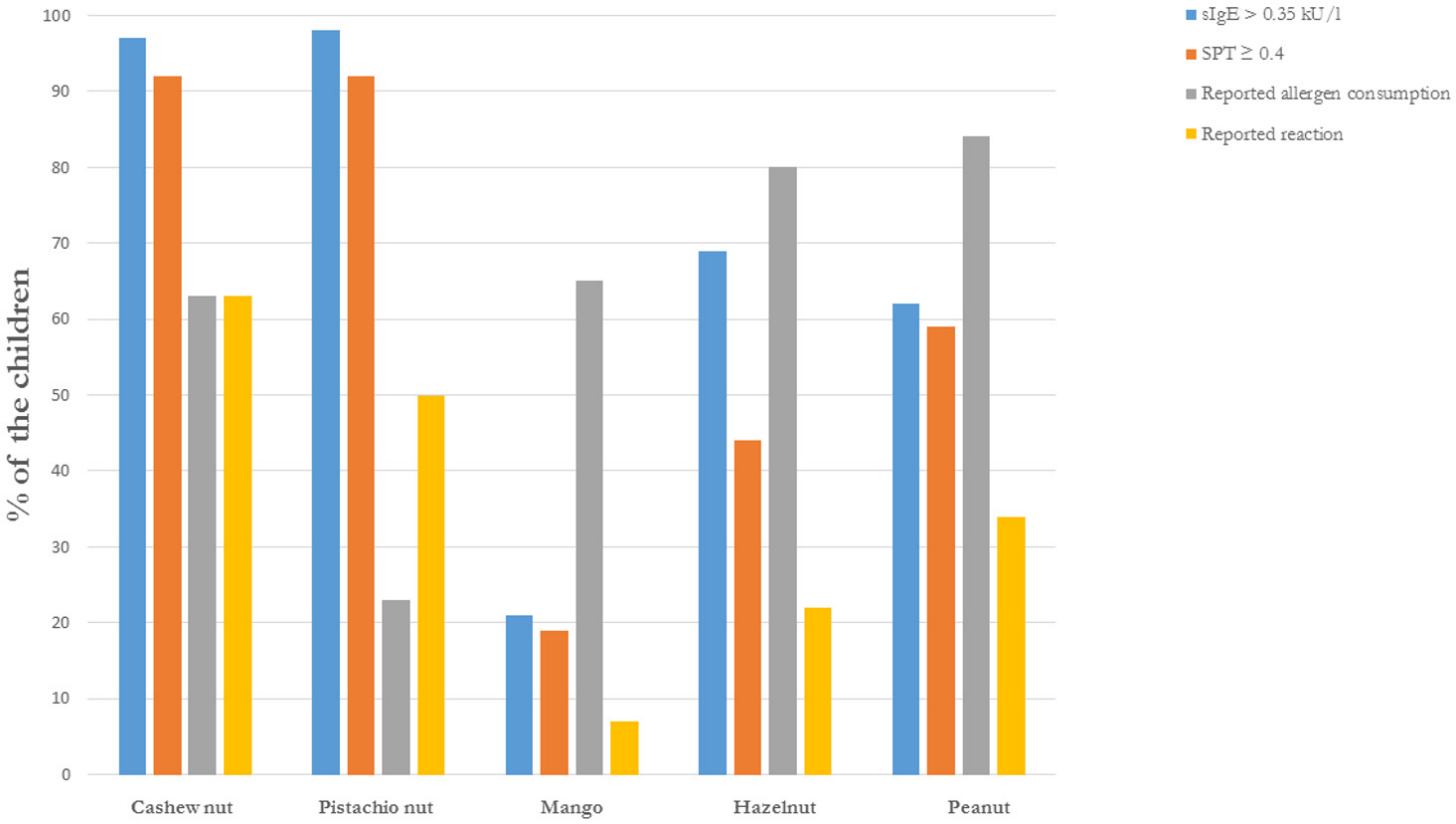
History and sensitisation to tested allergens. This figure shows the history and sensitisation to cashew nut and the history and co-sensitisation to pistachio nut, mango, hazelnut and peanut.

Twenty-three percent (42 of 179 children) reported pistachio nut consumption. 21 of 42 children (50%) reported allergic symptoms to pistachio nut. of these children. Mango was consumed by 116 of 179 children (65%) and allergy was reported in 8 of 116 children (7%). Hazelnuts were consumed by 143 of 179 children (80%) and 32 of these 143 children (22%) reported a hazelnut allergy. Peanuts were consumed by 151 of 179 children (84%) and peanut allergy was reported in 52 of these 151 children (34%) (see Fig 2).

### Sensitisation to cashew nut

173 children had a positive sIgE cashew value (> 0.35 kU/l) and 164 had a positive SPT (diameter ≥ 3 mm and HEP-index area ≥ 0.4). The median sIgE cashew was 3.72 kU/l (range 0-≥100 kU/l). The median HEP-index area of cashew SPT was 3.02 (range 0–15.16).

### Food challenge test

A total of 179 children were challenged with cashew nuts and 137 of the challenges were considered positive (76.5%), 36 negative (20.1%) and 6 undecided (3.4%). Most children experienced gastro-intestinal symptoms (nausea, vomiting, stomach pain and diarrhea), followed by oral allergy symptoms, skin symptoms (redness and itchiness), urticaria and angioedema (Table 5). A total of 49 (36%) of the children had an anaphylactic reaction as defined by the EAACI Guidelines for Food Allergy and Anaphylaxis (7). The most commonly observed type of anaphylactic reaction was a combination of skin and gastro-intestinal symptoms (Table 6). A total of 8 children (6%) with an positive reaction were treated with epinephrine. A single dose (0.15ml < 25 kg and 0.30ml >25 kg) of epinephrine was sufficient to treat the child. None of the children had a life-threatening reaction.

**Table 5:** Clinical symptoms during positive DBPCFC tests.

Symptoms	Number of children with positive DBPCFC test	
	**Total N = 137 (76.5%)**	
**Gastro-intestinal**	Number	%
Oral allergy	87	64
Nausea, stomach pain, vomiting, diarrhea	98	72
**Skin**		
Urticaria	29	21
Redness, itchiness	38	28
Angioedema	37	27
**Eye symptoms**	26	19
**Upper airway symptoms**	20	15
**Lower airway symptoms**	9	7
**Cardio-vascular symptoms**	0	0
**Indefinite symptoms**		
Change in behavior	18	13
Pallor/ feeling weak	9	7

**Table 6:** Anaphylactic reactions during positive DBPCFC tests.

**Anaphylaxis**	**Total N = 49/137 (36%)**
Skin and respiratory	3 (2%)
Skin and decrease of blood pressure*	0
Skin and gastro-intestinal	40 (30%)
Respiratory and decrease of blood pressure*	0
Respiratory and gastro-intestinal	6 (4%) **
Decrease of blood pressure and gastro-intestinal	0
Decrease of blood pressure > 30% SB	0

*Or associated symptoms such as syncope, incontinence and collapse

** Children had also skin symptoms.

**Fig 3:**
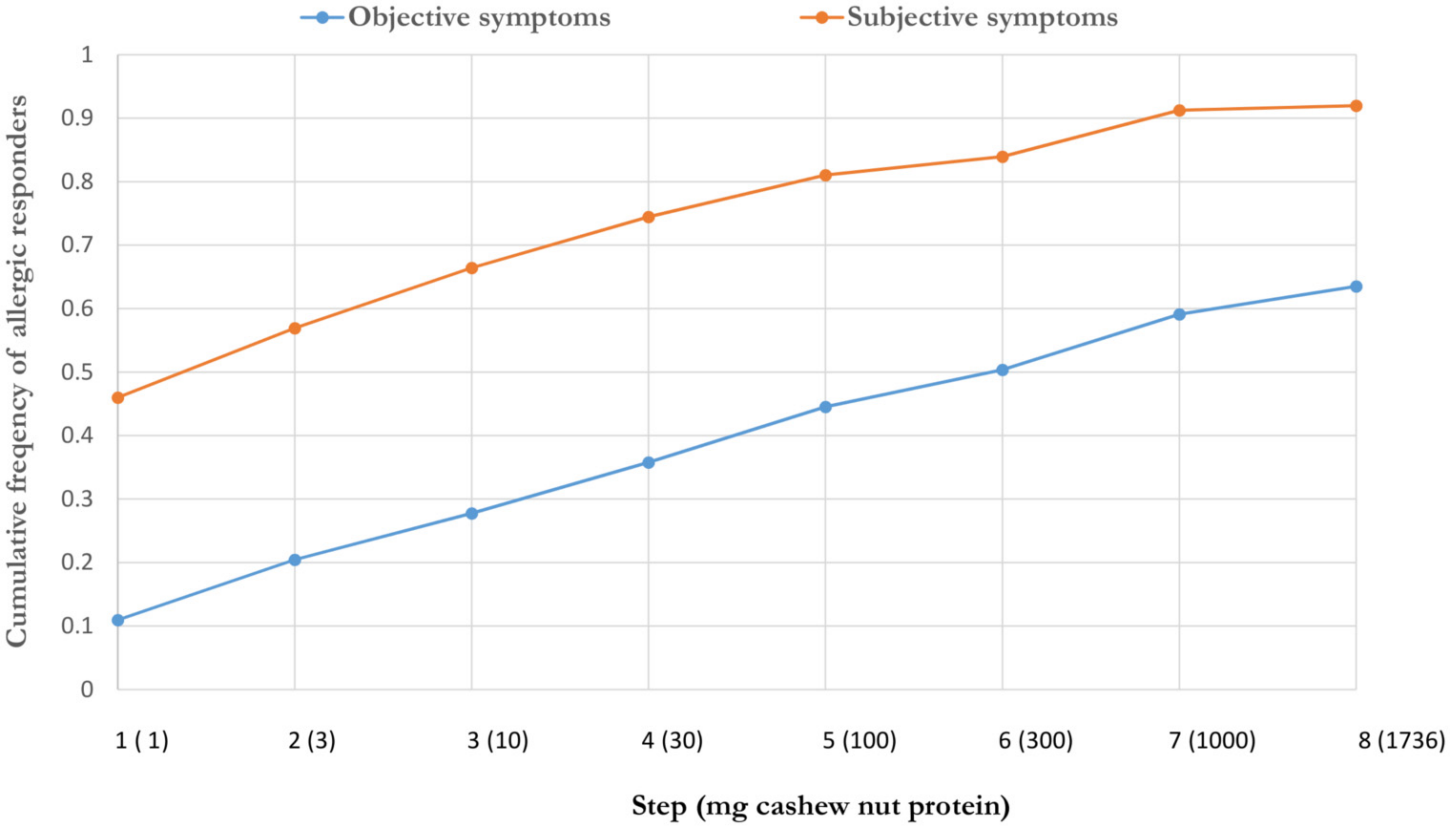
Threshold distribution curve for objective and subjective symptoms in cashew nut allergic children.

Only objective symptoms were seen in 16 children (12%), 47 children reported only subjective symptoms (34%) and 74 (54%) of the children showed both. After the first dose (1 mg cashew protein) 63 (46%) children experienced subjective symptoms and objective symptoms were observed after the first dose in 15 children (11%). Fig 3 shows the threshold distribution curve for objective and subjective symptoms. Anaphylaxis was observed in 23 (17%) children with the start of the reaction to the first dose. Cashew nut allergy could not be confirmed with the DBPCFC test in almost 20% of the children with a positive history.

### Co-sensitisation

In cashew nut sIgE-sensitised children, sIgE co-sensitisation to pistachio nuts was observed in 98% of the cases (169/173), to hazelnut in 69% of the cases (119/173), to peanut in 62% of the cases (107/173), to mango in 21% of the cases (37/173) and in 77% of the cases (134/173) to tree pollen. SPT cashew co-sensitisation to pistachio nut was seen in 92% (151/164). Lower percentages of SPT co-sensitisations were seen for hazelnut 71/163 (44%), to peanut 97/164 (59%) to mango 31/164 (19%), and to birch pollen 99/163 (61%).

## Discussion

Here, we present a diagnostic study in children sensitised to cashew nuts, carried out in three pediatric food allergy expertise centres in the Netherlands. We performed a DBPCFC test with cashew nut in this group, to measure the clinical relevance of sensitisation, to investigate the severity of the allergic reaction to cashew nut and the dosage of cashew nut to which they react.

More than 75% of the children sensitised to cashew nuts showed a clinical response in the DBPCFC test. This percentage is much higher than that observed in a previous other cashew nut study that analysed the clinical features of 42 children with a clinical history suggestive of cashew nut allergy, and a positive skin prick test (SPT) and/or a positive specific sIgE and/or a previous positive food challenge test [10]. Only in 8 (19%) of these children a cashew nut allergy could be confirmed with a positive challenge test. The percentage of a clinically relevant sensitisation to cashew nut in our study was also higher compared to the results of studies with other food allergens such as hazelnut. A study by Flinterman et al. showed that of the 28 children sensitised to hazelnut, a DBPCFC test could only confirm a hazelnut allergy in half of the patients [11]. During the challenge test in our study, most patients experienced gastro-intestinal symptoms, with skin manifestations as the second most prevalent symptom. This is in contrast to other studies on cashew-allergic patients in which skin symptoms were observed more frequently than respiratory and gastro-intestinal symptoms [10]. Sixty-three percent of the children tested reported a history of allergic reactions to cashew nuts in our study. However, a cashew nut allergy could not be confirmed with the DBPCFC test in almost 20% of the children with a positive history. Half of these children experienced the last allergic symptoms to cashew nut between one month and two years ago. A negative oral food challenge test after positive testing and/or positive history is reported between 9% and 38% for peanut allergy [12–16]. Children with a positive history and negative testing may have outgrown their allergy or may have an unreliable history. In addition personal co-factors or differences in exposure may account for this discrepancy. Accidental ingestion of cashew nut is not very likely as they are incorporated in products in an unrecognisable form less often than peanut or hazelnut.

With the first dosage of only 1 mg of cashew protein, 46% of the children experienced subjective and 11% objective symptoms. The food allergy threshold study by Blom et al. with 363 DBPCFC tests showed that the number of patients with any type of symptoms caused by 1 mg cashew nut-, hazelnut-, egg-, milk- and peanut protein varied between 5 and 20% [17]. The number of patients with objective symptoms to 1 mg hazelnut protein was reported in 10% of the patients and was comparable with our results for cashew nut. The number of patients reported with objective symptoms to 1 mg egg-, milk- and peanut protein was lower compared with cashew nut in our study. This confirms the potency and thus the potentially dangerous nature of cashew nut compared to other allergens.

Almost 40% of the children in our study showed anaphylactic reactions and 6% of these children was treated with epinephrine. Anaphylaxis was observed in 17% of the children with the start of the allergic reaction to the first dose (1 mg cashew nut protein). A previous threshold study demonstrated that approximately 5% of 257 peanut allergic patients reacted to 1 mg peanut protein with severe symptoms [18]. Therefore, our study supports previous observations, showing that minimal amounts of cashew nut are sufficient to cause these severe allergic reactions.

Cashew nut, pistachio nut and mango belong to the *Anacardiaceae* family and are thus botanically related. In line with previous reports, this study shows a high rate of co-sensitisation between cashew nuts and pistachio nuts in SPT and sIgE (respectively 92% and 98%) [19–22]. Almost 50% (21/42) of our pistachio nut sensitized and exposed children reported allergic reactions to pistachio nuts.

Many cashew nut and pistachio nut sensitised children reported no consumption of cashew nut and/or pistachio nut. In most cases, these children were previously advised to eliminate cashew nuts and also pistachio nuts from the diet because of the possibility of cross-reactions. We advise, however, in these cases to perform a DBPCFC test to avoid unnecessary eliminations.

Mango is also botanically related to cashew nut, but our study shows only 19% co-sensitisation with mango in cashew positive SPT children and 21% in sIgE positive children. In this study, almost all children have consumed mango and only 7% reported a history of reactions due to the consumption of mango. Cross-reactivity has not been reported between cashew nut and mango [1] and oral challenges with mango are necessary to confirm the histories we obtained.

### Strengths and weaknesses of this study

The strength of this study was the prospective multicentre study design and its size compared to previous studies on cashew nut allergy [1]. This relatively large number of children enabled us to estimate the rate of clinical reactions in sensitised children and to determine the severity of cashew nut allergy. All children underwent a DBPCFC test with cashew nut and thus, the diagnosis was based on this gold standard to establish food allergy, and consequently the clinical relevance of sensitisation and potency of the cashew nut allergen could be accurately examined. A limitation of this study was that we were unable to use the well-accepted scoring system to assess DBPCFC tests as proposed by a PRACTALL consensus group, as this was published after the start of the study [23]. Furthermore, there might be a selection bias in this study because a lot of children or parents of children refused to participate in this study because of e.g. fear for severe allergic reactions during the DBPCFC test. However, there was also a large group of children with an unknown history of cashew nut ingestion and among this group were children with a severe cashew nut allergy. Furthermore, many children experienced an anaphylactic reaction in this study. Therefore, the selection bias seems to be small.

## Conclusion

This is the largest prospective clinical study reported in children, sensitised to cashew nut so far. The study demonstrates a high percentage of clinical reactivity to cashew nut in sensitised children. Cashew nuts may cause severe allergic reactions, including anaphylaxis. A minimal amount of cashew nut (1 mg comparable with 1/100 part of a cashew nut) may be sufficient to cause clinical symptoms.

## Supporting Information

S1 TREND Checklist (PDF)

S1 Database IDEAL-Study (PDF)

S1 Study protocol (DOC)

## Abbreviations

DBPCFC: Double-blind placebo-controlled food challenge
HEP: Histamine equivalent prick
sIgE: Specific IgE
SPT: Skin prick test
