# Supplementary material for: Multicentre Double-Blind Placebo-Controlled Food Challenge Study in Children Sensitised to Cashew Nut
Source: PLoS One. 2016 Mar 11;11(3):e0151055. doi: 10.1371/journal.pone.0151055 (PMC4788393; doi:10.1371/journal.pone.0151055)
Supplement: S1 Database IDEAL-Study — (PDF) [file pone.0151055.s002.pdf]

Data IDEAL-study Trial number NTR3572

	Patient_ID	Age	Gender	Hay_Fever	Asthma	Eczema	Atopy	Outcome_challange	sIgE_Cashew	sIgE_Pistachio	SPT_Cashew	SPT_Pistachio	Anaphylaxis
1	1110001	11	Female	Yes	No	No	Yes	Negative	15,70	20,60	1,82	1,91	No reaction
2	1110002	9	Female	Yes	No	Yes	Yes	Positive	3,18	5,89	4,60	2,97	Anaphylaxis
3	1110004	5	Male	No	No	No	No	Positive	8,96	13,70	2,36	,50	Mild reaction
4	1110005	8	Female	Yes	No	No	Yes	Positive	23,70	29,20	4,48	1,77	Mild reaction
5	1110006	5	Male	Yes	No	Yes	Yes	Negative	,85	,60	,37	,14	No reaction
6	1110007	13	Male	Yes	No	Yes	Yes	Negative	1,30	1,35	,00	,00	No reaction
7	1110008	11	Female	No	Yes	Yes	Yes	Negative	,00	,00	,49	1,14	No reaction
8	1110009	14	Male	Yes	Yes	Yes	Yes	Positive	21,80	26,90	1,25	2,04	Mild reaction
9	1110010	11	Female	No	No	No	No	Positive	10,90	8,39	1,11	3,14	Anaphylaxis
10	1110011	12	Male	Yes	Yes	Yes	Yes	Positive	11,10	19,90	3,28	4,77	Mild reaction
11	1110012	4	Female	No	No	Yes	Yes	Positive	13,00	18,50	3,98	3,14	Mild reaction
12	1110013	8	Female	No	Yes	Yes	Yes	Positive	2,63	6,63	1,68	1,37	Mild reaction
13	1110014	17	Female	Yes	Yes	Yes	Yes	Negative	5,28	11,50	,59	,00	No reaction
14	1110015	9	Male	Yes	Yes	Yes	Yes	Positive	38,60	37,60	2,11	7,48	Mild reaction
15	1110016	13	Female	Yes	No	No	Yes	Negative	2,16	1,10	,39	,27	No reaction
16	1110019	7	Female	Yes	Yes	Yes	Yes	Negative	,65	,00	,30	,99	No reaction
17	1110020	4	Female	No	No	No	No	Positive	6,03	7,92	3,21	1,44	Anaphylaxis
18	1110021	6	Male	No	Yes	Yes	Yes	Negative	3,76	3,55	4,37	,19	No reaction
19	1110022	16	Female	Yes	Yes	No	Yes	Undetermined	,38	,59	3,28	5,68	No reaction
20	1110023	14	Male	Yes	No	Yes	Yes	Positive	1,25	2,33	3,38	6,04	Anaphylaxis
21	1110024	9	Female	No	Yes	No	Yes	Negative	1,17	2,36	3,19	,97	No reaction
22	1110025	6	Female	No	No	No	No	Positive	1,79	4,61	5,89	1,35	Mild reaction
23	1110027	8	Male	No	Yes	Yes	Yes	Positive	27,00	79,50	1,36	,78	Mild reaction
24	1110028	3	Female	No	No	Yes	Yes	Positive	100,00	100,00	5,54	9,39	Anaphylaxis
25	1110029	11	Male	Yes	Yes	Yes	Yes	Positive	7,39	4,37	5,13	8,29	Mild reaction
26	1110030	10	Female	No	No	No	No	Positive	44,70	47,10	9,33	9,19	Anaphylaxis
27	1110031	9	Male	Yes	Yes	No	Yes	Positive	13,10	29,80	2,16	2,75	Anaphylaxis
28	1110032	6	Female	No	No	Yes	Yes	Positive	10,80	16,20	,65	2,31	Anaphylaxis
29	1110033	2	Male	No	No	No	No	Negative	,00	,00	14,25	,00	No reaction
30	1110034	4	Male	Yes	No	Yes	Yes	Positive	1,38	3,81	3,14	4,92	Mild reaction
31	1110035	5	Female	No	No	No	No	Positive	3,06	5,16	2,28	6,29	Anaphylaxis
32	1110037	13	Female	No	No	No	No	Positive	6,42	10,90	2,49	1,95	Mild reaction
33	1110038	9	Male	Yes	Yes	Yes	Yes	Positive	,46	,27	3,57	,91	Anaphylaxis
34	1110039	3	Male	No	No	Yes	Yes	Positive	47,40	51,40	6,01	12,30	Anaphylaxis
35	1110040	2	Male	No	Yes	Yes	Yes	Negative	5,11	4,51	,53	,71	No reaction
36	1110041	7	Male	Yes	No	Yes	Yes	Negative	,63	,41	,50	2,59	No reaction
37	1110042	2	Male	No	No	No	No	Negative	2,14	2,50	1,32	,84	No reaction
38	1110043	15	Female	Yes	No	Yes	Yes	Negative	,63	,97	,11	,13	No reaction
39	1110045	9	Male	Yes	No	Yes	Yes	Positive	1,02	1,74	,89	1,13	Anaphylaxis
40	1110046	17	Female	No	No	No	No	Positive	,57	,90	3,15	2,64	Anaphylaxis
41	1110048	15	Male	No	No	Yes	Yes	Positive	,41	,37	,90	,54	Mild reaction
42	1110049	5	Female	No	No	No	No	Positive	7,28	11,50	3,17	1,91	Mild reaction
43	1110052	13	Male	Yes	Yes	No	Yes	Positive	3,72	8,28	2,35	8,85	Mild reaction
44	1110053	3	Male	No	Yes	Yes	Yes	Positive	51,90	61,80	5,54	2,19	Mild reaction
45	1110054	9	Male	Yes	Yes	No	Yes	Positive	22,10	36,30	4,53	4,23	Anaphylaxis
46	1110055	6	Male	Yes	Yes	Yes	Yes	Positive	10,80	16,20	1,48	2,43	Anaphylaxis
47	1110056	4	Male	No	Yes	Yes	Yes	Positive	12,70	10,70	5,48	2,98	Anaphylaxis
48	1110057	6	Female	No	No	No	No	Positive	9,00	7,17	2,61	3,97	Mild reaction
49	1110058	4	Female	Yes	Yes	Yes	Yes	Positive	48,70	92,90	2,65	2,63	Anaphylaxis
50	1110059	4	Female	No	No	Yes	Yes	Positive	3,10	3,06	5,43	3,07	Mild reaction
51	1110060	13	Female	No	No	No	No	Positive	9,11	7,98	3,31	2,73	Mild reaction
52	1110061	10	Female	Yes	Yes	Yes	Yes	Positive	1,01	1,99	1,41	2,02	Mild reaction
53	1110062	6	Male	No	No	Yes	Yes	Positive	30,20	44,50	9,41	5,39	Mild reaction
54	1110063	8	Male	No	Yes	No	Yes	Positive	2,57	1,54	2,41	2,25	Mild reaction
55	1110064	12	Male	No	No	No	No	Positive	7,65	7,25	1,95	1,66	Mild reaction
56	1110065	7	Male	No	No	No	No	Positive	,68	,91	5,36	2,80	Anaphylaxis
57	1110066	4	Female	.	No	Yes	Yes	Positive	2,00	3,02	1,86	1,89	Anaphylaxis
58	1110067	5	Male	No	No	Yes	Yes	Positive	1,04	,83	,58	,21	Mild reaction
59	1110069	5	Female	No	No	No	No	Positive	2,76	2,13	4,95	5,00	Anaphylaxis
60	1110070	14	Male	No	No	Yes	Yes	Positive	1,94	3,93	3,87	7,67	Mild reaction
61	1110071	16	Male	No	No	No	No	Positive	7,08	14,40	3,34	2,42	Mild reaction
62	1110072	2	Male	No	No	No	No	Positive	3,23	4,80	3,93	5,58	Mild reaction
63	1110073	3	Male	No	No	Yes	Yes	Positive	1,69	1,03	,39	,71	Mild reaction
64	1110075	3	Male	No	No	No	No	Negative	,48	,21	,46	,26	No reaction
65	1110076	4	Female	Yes	No	No	Yes	Positive	,73	,71	5,80	2,26	Anaphylaxis
66	1110079	10	Male	No	No	No	No	Positive	100,00	82,00	2,50	2,05	Mild reaction
67	2220001	8	Male	No	Yes	No	Yes	Positive	4,49	8,79	4,34	3,74	Mild reaction
68	2220002	8	Male	Yes	Yes	Yes	Yes	Undetermined	1,62	3,49	2,31	7,18	No reaction
69	2220003	6	Male	No	No	No	No	Negative	1,57	1,46	,82	2,08	No reaction

	Patient_ID	Age	Gender	Hay_Fever	Asthma	Eczema	Atopy	Outcome_challange	sIgE_Cashew	sIgE_Pistachio	SPT_Cashew	SPT_Pistachio	Anaphylaxis
70	2220004	11	Female	Yes	No	No	Yes	Positive	13,20	26,40	5,02	8,72	Anaphylaxis
71	2220005	6	Male	No	No	Yes	Yes	Undetermined	,83	,98	3,37	2,40	No reaction
72	2220006	14	Male	No	Yes	Yes	Yes	Positive	100,00	100,00	9,66	9,37	Mild reaction
73	2220007	13	Male	No	Yes	No	Yes	Positive	1,77	3,98	7,48	5,13	Mild reaction
74	2220008	10	Female	Yes	Yes	No	Yes	Positive	4,81	9,33	1,16	2,70	Mild reaction
75	2220009	12	Male	Yes	No	No	Yes	Positive	37,70	50,10	1,37	1,96	Mild reaction
76	2220010	7	Male	Yes	Yes	Yes	Yes	Positive	3,53	5,12	3,38	2,80	Anaphylaxis
77	2220011	10	Male	No	No	Yes	Yes	Positive	1,08	1,08	4,80	2,88	Mild reaction
78	2220012	12	Male	Yes	Yes	Yes	Yes	Positive	100,00	100,00	1,16	2,52	Mild reaction
79	2220013	8	Female	Yes	No	Yes	Yes	Positive	,39	,93	2,68	1,65	Mild reaction
80	2220014	14	Male	No	No	No	No	Negative	17,80	12,10	2,73	2,40	No reaction
81	2220015	7	Male	No	No	No	No	Positive	6,55	5,89	3,50	5,50	Mild reaction
82	2220016	8	Male	Yes	Yes	No	Yes	Positive	41,20	44,80	5,61	5,89	Anaphylaxis
83	2220017	4	Female	No	No	Yes	Yes	Negative	2,70	2,42	1,24	,80	No reaction
84	2220018	6	Male	Yes	No	No	Yes	Positive	32,00	21,30	3,57	3,97	Mild reaction
85	2220019	8	Male	No	Yes	No	Yes	Positive	4,62	5,72	6,51	8,80	Mild reaction
86	2220020	12	Male	No	No	No	No	Negative	10,50	7,29	,00	1,47	No reaction
87	2220021	8	Female	No	Yes	No	Yes	Positive	8,75	9,29	14,36	12,00	Anaphylaxis
88	2220022	7	Female	Yes	No	Yes	Yes	Positive	1,56	2,41	8,76	6,26	Mild reaction
89	2220023	10	Male	Yes	Yes	Yes	Yes	Positive	21,20	30,10	2,94	3,77	Mild reaction
90	2220024	14	Male	No	No	No	No	Positive	15,20	11,70	6,85	9,35	Anaphylaxis
91	2220025	9	Male	No	Yes	No	Yes	Positive	82,00	100,00	4,25	3,91	Mild reaction
92	2220026	15	Male	Yes	Yes	Yes	Yes	Positive	3,58	5,26	4,76	1,64	Mild reaction
93	2220027	10	Male	Yes	Yes	No	Yes	Negative	5,48	5,05	1,47	1,37	No reaction
94	2220028	6	Female	Yes	No	No	Yes	Positive	17,50	25,80	13,00	7,98	Mild reaction
95	2220029	6	Female	No	No	No	No	Positive	9,34	10,00	3,29	5,23	Mild reaction
96	2220033	3	Male	Yes	No	No	Yes	Negative	3,01	3,66	,00	,77	No reaction
97	2220034	14	Female	Yes	Yes	Yes	Yes	Positive	8,29	13,40	10,70	12,19	Mild reaction
98	2220035	7	Male	No	No	No	No	Negative	,97	1,16	3,38	,00	No reaction
99	2220036	10	Female	Yes	Yes	Yes	Yes	Undetermined	4,33	10,20	2,70	3,93	No reaction
100	2220037	11	Male	Yes	No	No	Yes	Negative	2,97	7,51	4,22	2,66	No reaction
101	2220038	12	Female	Yes	No	Yes	Yes	Positive	16,90	19,60	1,90	6,77	Mild reaction
102	2220039	15	Male	No	No	No	No	Positive	4,76	8,60	4,68	5,47	Mild reaction
103	2220040	6	Male	No	No	No	No	Negative	,97	,97	,00	,71	No reaction
104	2220041	10	Female	No	No	No	No	Positive	,13	1,02	1,38	1,08	Anaphylaxis
105	2220042	9	Female	Yes	Yes	Yes	Yes	Positive	2,59	1,80	5,75	4,41	Anaphylaxis
106	2220043	10	Male	No	No	Yes	Yes	Positive	8,17	9,01	6,02	4,39	Mild reaction
107	2220044	6	Male	No	No	Yes	Yes	Negative	2,05	2,25	99,00	99,00	No reaction
108	2220045	13	Male	Yes	No	Yes	Yes	Positive	8,54	17,10	5,95	6,45	Mild reaction
109	2220048	7	Male	Yes	Yes	Yes	Yes	Positive	17,30	15,10	12,92	7,62	Mild reaction
110	2220052	7	Male	Yes	Yes	Yes	Yes	Negative	,89	,85	,00	,82	No reaction
111	2220053	5	Male	Yes	No	No	Yes	Positive	50,70	52,80	4,46	4,56	Anaphylaxis
112	2220055	5	Male	No	No	No	No	Positive	2,22	4,82	5,52	5,11	Anaphylaxis
113	2220057	6	Male	Yes	No	No	Yes	Positive	3,89	10,80	1,31	2,36	Mild reaction
114	3330001	13	Male	Yes	No	No	Yes	Positive	,92	1,83	9,60	10,73	Mild reaction
115	3330002	12	Male	Yes	No	Yes	Yes	Positive	3,53	7,92	2,90	3,97	Mild reaction
116	3330003	12	Male	Yes	No	No	Yes	Negative	,78	1,28	1,32	2,36	No reaction
117	3330004	6	Male	Yes	No	Yes	Yes	Negative	,51	,31	,37	,27	No reaction
118	3330005	6	Female	Yes	Yes	Yes	Yes	Positive	,59	,91	1,88	1,15	Anaphylaxis
119	3330006	15	Male	Yes	No	No	Yes	Negative	1,13	2,23	3,22	3,59	No reaction
120	3330007	14	Female	Yes	Yes	No	Yes	Negative	,52	,40	,28	,00	No reaction
121	3330008	4	Female	No	Yes	No	Yes	Positive	58,30	45,00	1,74	1,53	Mild reaction
122	3330009	9	Male	Yes	No	No	Yes	Positive	,83	1,36	3,53	2,40	Mild reaction
123	3330010	16	Male	Yes	No	No	Yes	Positive	9,90	2,02	3,46	2,61	Mild reaction
124	3330011	15	Male	No	No	Yes	Yes	Positive	100,00	100,00	5,64	9,91	Mild reaction
125	3330012	8	Male	Yes	No	Yes	Yes	Undetermined	11,80	16,90	6,27	6,39	No reaction
126	3330013	7	Female	Yes	No	No	Yes	Positive	,00	,00	,54	,42	Mild reaction
127	3330014	9	Male	Yes	No	Yes	Yes	Positive	10,00	14,10	1,73	,72	Anaphylaxis
128	3330015	4	Female	No	No	No	No	Positive	32,00	22,40	,82	4,22	Mild reaction
129	3330016	5	Male	Yes	No	Yes	Yes	Positive	1,50	2,02	6,75	7,42	Mild reaction
130	3330017	13	Male	No	No	No	No	Positive	3,81	5,15	5,45	3,27	Mild reaction
131	3330018	10	Male	No	No	No	No	Positive	100,00	100,00	6,01	10,93	Anaphylaxis
132	3330019	10	Male	Yes	No	No	Yes	Negative	1,52	2,26	1,78	,79	No reaction
133	3330020	10	Female	Yes	No	No	Yes	Positive	,43	1,14	,93	,94	Mild reaction
134	3330021	12	Male	No	No	No	No	Positive	1,13	1,17	2,09	2,44	Mild reaction
135	3330023	16	Male	Yes	No	No	Yes	Positive	3,31	7,35	6,36	7,46	Anaphylaxis
136	3330024	10	Female	Yes	No	No	Yes	Positive	,90	3,66	10,75	4,72	Anaphylaxis
137	3330025	10	Male	No	Yes	No	Yes	Positive	48,10	41,90	3,04	3,70	Mild reaction
138	3330027	7	Male	Yes	No	No	Yes	Positive	32,60	30,90	5,33	5,44	Mild reaction

	Patient_ID	Age	Gender	Hay_Fever	Asthma	Eczema	Atopy	Outcome_challange	sIgE_Cashew	sIgE_Pistachio	SPT_Cashew	SPT_Pistachio	Anaphylaxis
139	3330028	8	Male	Yes	No	No	Yes	Negative	,72	,60	,43	,00	No reaction
140	3330029	15	Male	Yes	No	Yes	Yes	Positive	,25	,22	,67	,08	Mild reaction
141	3330030	14	Female	No	Yes	No	Yes	Positive	4,66	10,30	5,46	7,86	Mild reaction
142	3330031	7	Female	No	No	No	No	Positive	3,28	7,76	15,16	3,24	Anaphylaxis
143	3330032	5	Female	No	No	Yes	Yes	Positive	2,33	2,94	,77	,15	Mild reaction
144	3330033	15	Female	Yes	No	No	Yes	Positive	32,90	47,20	5,29	,00	Anaphylaxis
145	3330034	2	Male	No	Yes	No	Yes	Positive	2,12	1,57	3,09	1,36	Mild reaction
146	3330035	10	Female	No	No	No	No	Negative	5,26	2,75	,87	,91	No reaction
147	3330036	10	Male	No	No	No	No	Negative	,43	,46	,30	,00	No reaction
148	3330037	15	Female	No	No	No	No	Positive	1,08	1,03	1,11	2,59	Anaphylaxis
149	3330038	11	Female	Yes	No	No	Yes	Positive	,47	,84	1,22	1,57	Mild reaction
150	3330039	3	Female	Yes	No	Yes	Yes	Positive	48,40	42,40	4,53	7,54	Mild reaction
151	3330040	7	Male	No	No	No	No	Positive	2,35	3,82	4,95	1,93	Mild reaction
152	3330041	9	Female	Yes	No	No	Yes	Undetermined	10,10	16,00	1,62	6,76	No reaction
153	3330042	5	Female	Yes	No	No	Yes	Positive	18,40	20,10	2,21	,00	Anaphylaxis
154	3330043	11	Female	No	Yes	Yes	Yes	Positive	22,50	30,20	5,49	4,66	Mild reaction
155	3330044	11	Male	Yes	No	No	Yes	Positive	2,33	2,67	3,53	,00	Anaphylaxis
156	3330045	10	Male	Yes	No	No	Yes	Positive	12,10	42,50	1,46	2,83	Anaphylaxis
157	3330047	11	Female	Yes	No	Yes	Yes	Positive	5,13	10,90	2,29	1,55	Mild reaction
158	3330048	9	Female	Yes	No	Yes	Yes	Positive	5,04	3,85	1,78	4,14	Mild reaction
159	3330049	7	Male	No	No	No	No	Positive	2,17	3,13	4,77	2,07	Anaphylaxis
160	3330050	4	Female	Yes	Yes	No	Yes	Positive	2,55	5,18	3,73	3,27	Anaphylaxis
161	3330051	11	Male	Yes	Yes	No	Yes	Positive	11,20	8,96	5,10	9,19	Anaphylaxis
162	3330052	10	Female	Yes	Yes	No	Yes	Positive	49,00	34,20	1,16	,99	Mild reaction
163	3330053	11	Male	No	No	No	No	Negative	,21	,46	2,92	1,16	No reaction
164	3330054	4	Female	Yes	No	Yes	Yes	Positive	17,70	10,50	3,86	5,22	Mild reaction
165	3330055	5	Female	Yes	No	No	Yes	Positive	21,10	37,30	,77	3,77	Anaphylaxis
166	3330056	14	Male	Yes	No	No	Yes	Positive	1,44	1,75	3,86	3,75	Mild reaction
167	3330057	13	Male	Yes	Yes	No	Yes	Positive	11,90	23,20	1,47	1,13	Anaphylaxis
168	3330058	8	Female	Yes	No	No	Yes	Positive	1,41	,65	2,54	,72	Mild reaction
169	3330059	6	Male	No	No	No	No	Positive	3,22	9,20	3,00	3,50	Mild reaction
170	3330060	12	Male	Yes	Yes	No	Yes	Positive	,86	1,70	2,43	1,07	Mild reaction
171	3330061	16	Female	No	No	No	No	Positive	1,33	1,67	2,68	2,04	Anaphylaxis
172	3330062	6	Female	Yes	Yes	No	Yes	Positive	37,40	68,30	3,37	7,23	Anaphylaxis
173	3330063	9	Female	Yes	No	No	Yes	Positive	7,89	18,50	3,88	1,28	Mild reaction
174	3330064	12	Male	No	No	No	No	Positive	2,02	2,44	3,98	1,81	Mild reaction
175	3330065	2	Female	Yes	No	No	Yes	Positive	1,71	,74	1,49	,00	Mild reaction
176	3330066	7	Male	Yes	No	No	Yes	Positive	87,90	100,00	2,33	2,92	Mild reaction
177	3330067	2	Male	Yes	No	No	Yes	Positive	5,83	14,50	4,74	4,47	Mild reaction
178	3330068	11	Female	No	No	No	No	Positive	,42	,93	1,70	1,47	Anaphylaxis
179	3330070	14	Male	No	No	No	No	Negative	2,76	2,17	,15	,49	No reaction
