# Supplementary material for: Multicentre Double-Blind Placebo-Controlled Food Challenge Study in Children Sensitised to Cashew Nut
Source: PLoS One. 2016 Mar 11;11(3):e0151055. doi: 10.1371/journal.pone.0151055 (PMC4788393; doi:10.1371/journal.pone.0151055)
Supplement: S1 Study protocol — (DOC) [file pone.0151055.s003.doc]

**Clinical Trial Protocol**

**Improvement of Diagnostic mEthods for ALlergy assessment. Cashew allergy in children as a showcase (IDEAL study)**

**Coordinating investigator**: N.W. de Jong PhD, Erasmus MC Rotterdam, Netherlands

**Co-investigators**: R. Gerth van Wijk, MD PhD, Erasmus MC Rotterdam, Netherlands

A.E.J. Dubois MD PhD, UMC Groningen, Netherlands

H. de Groot MD PhD, Reinier de Graaf Gasthuis, Delft, Netherlands

H.J. Wichers PhD, Wageningen Universiteit, Netherlands

H.F.J. Savelkoul PhD, Wageningen Universiteit, Netherlands

E.W. Steyerberg PhD, Erasmus MC Rotterdam, Netherlands

Protocol author: N.W. de Jong PhD

Protocol version: nr 1, February 17, 2012

Anticipated start date: May 1 2012

| 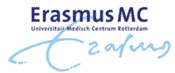 | [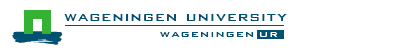](http://www.wageningenuniversity.nl/UK/) |
| --- | --- |

Improvement of Diagnostic mEthods for ALlergy assessment. Cashew allergy in children as a showcase.

| Protocol ID | IDEAL |
| --- | --- |
| Short title | Improvement of diagnostic methods for allergy assessment |
| Coordinating Investigator; Project leader: | dr. N.W. de Jong BE*d*.  Erasmus MC  Afdeling Interne Geneeskunde  Sectie Allergologie, gK 323  Postbus 2040  3000 CA Rotterdam, Nederland  +31 10 7034080  [n.w.dejong@erasmusmc.nl](mailto:n.w.dejong@erasmusmc.nl) |
| Sponsor | Erasmus MC, Rotterdam |
| Financial support | NWO-Technologiestichting STW-Open Technologie Programma OTP |
| Co-investigators | Prof. dr. R. Gerth van Wijk  Erasmus MC  Dept. Of Internal Medicine  section Allergology, gK 323  PO Box 2040  3000 CA Rotterdam, Netherlands  +31 10 7035970  R.Gerthvanwijk@erasmusmc.nl |

|  | Prof. dr. H.J. Wichers  Wageningen Universiteit en Research Centrum  PO Box 17  6700 AA Wageningen, Netherlands  +31 317 480175  harry.wichers@wur.nl |
| --- | --- |

|  | Prof. dr. ir. H.F.J. Savelkoul  Wageningen Universiteit  Celbiology & Immunology  Marijkeweg 40  6709 PG Wageningen, Netherlands  +31 317 483 925  huub.savelkoul@wur.nl |
| --- | --- |

|  | Prof. dr. A.E.J. Dubois  Universitair Medisch Centrum Groningen  Hanzeplein 1 PO Box 30.001 9700 RB Groningen, Netherlands  +31 50 3612748  a.e.j.dubois@bkk.umcg.nl |
| --- | --- |

|  | Prof. dr. E.W. Steyerberg  Erasmus MC  Dept. Medische Besliskunde  PO Box 2040  3000 CA Rotterdam, Netherlands  +31 10 7038470  [e.steyerberg@erasmusmc.nl](mailto:e.steyerberg@erasmusmc.nl) |
| --- | --- |
|  | dr. H. de Groot  Reinier de Graaf Gasthuis  PO Box 5011  2600 GA Delft, Netherlands  +31 152603060  GrootH@rdgg.nl |

| Independent Physician | Prof. dr. N. Hartwig  Erasmus MC  Dept. Of Infectious Diseases/Immunology sK 2145  PO Box 2040  3000 CA Rotterdam, Netherlands  +31 10 7034080 |
| --- | --- |
| Laboratory sites | Wageningen Universiteit en Research Centrum |

**PROTOCOL SIGNATURE SHEET**

| Name | Signature | Date |
| --- | --- | --- |
| Head of Department Erasmus MC:  Prof. dr. R. Gerth van Wijk |  |  |
| Coordinating Investigator/Project leader/Principal Investigator:  Dr. N.W. de Jong |  |  |

**Table of contents**

[Co-investigators 2](#__RefHeading___Toc317435624)

[Summary 8](#__RefHeading___Toc317435625)

[1 Introduction and rationale 9](#__RefHeading___Toc317435626)

[1.1. Current status of allergy diagnosis 9](#__RefHeading___Toc317435627)

[1.2. Cashew nut allergy as a model to evaluate diagnostic procedures 9](#__RefHeading___Toc317435628)

[1.3. Preliminary results 9](#__RefHeading___Toc317435629)

[2. Optimization of the diagnosis of cashew nut allergy 11](#__RefHeading___Toc317435630)

[2.1. Components of diagnostic procedures 11](#__RefHeading___Toc317435631)

[2.2. Cross reactivity between cashew nuts and other allergens 12](#__RefHeading___Toc317435632)

[3. Study objectives 13](#__RefHeading___Toc317435633)

[3.1. Primary objectives 13](#__RefHeading___Toc317435634)

[3.2. Secondary objective 13](#__RefHeading___Toc317435635)

[4. Investigational plan 14](#__RefHeading___Toc317435636)

[4.1. Study design 14](#__RefHeading___Toc317435637)

[4.2. Existing infrastructure 14](#__RefHeading___Toc317435638)

[5. Population 16](#__RefHeading___Toc317435639)

[5.1. Inclusion criteria 16](#__RefHeading___Toc317435640)

[5.2. Exclusion criteria 16](#__RefHeading___Toc317435641)

[5.3. Sample size calculation 16](#__RefHeading___Toc317435642)

[6. Methods 17](#__RefHeading___Toc317435643)

[6.1. Visit schedule and assessments 17](#__RefHeading___Toc317435644)

[6.2. Patient numbering 17](#__RefHeading___Toc317435645)

[6.3. Data to be collected/assessments 17](#__RefHeading___Toc317435646)

[6.3.1. History 17](#__RefHeading___Toc317435647)

[Patient demographics/other baseline characteristics 17](#__RefHeading___Toc317435648)

[6.3.3. FAQLQ Questionnaire 18](#__RefHeading___Toc317435649)

[6.3.4. Physical examination 18](#__RefHeading___Toc317435650)

[6.4. Time table 18](#__RefHeading___Toc317435651)

[6.5. Skin prick tests 18](#__RefHeading___Toc317435652)

[6.6. Double blind placebo controlled food challenge (visit 2 and 3) 19](#__RefHeading___Toc317435653)

[6.6.1. Background 19](#__RefHeading___Toc317435654)

[6.6.2. Patients 20](#__RefHeading___Toc317435655)

[6.6.3. Performance 20](#__RefHeading___Toc317435656)

[6.6.4. Randomization, blinding and preparation 21](#__RefHeading___Toc317435657)

[6.6.5. Assessment protocol for the outcome 22](#__RefHeading___Toc317435658)

[6.6.6. Results, telephone contact 24](#__RefHeading___Toc317435659)

[6.7. Blood collection 24](#__RefHeading___Toc317435660)

[6.7.1.IgE 24](#__RefHeading___Toc317435661)

[6.7.2.BAT 24](#__RefHeading___Toc317435662)

[6.7.3. RBL 24](#__RefHeading___Toc317435663)

[6.8. Withdrawal of individual subjects 24](#__RefHeading___Toc317435664)

[7. Data collection and statistical analysis 25](#__RefHeading___Toc317435665)

[8. Ethical considerations 26](#__RefHeading___Toc317435666)

[8.1. Regulation statement 26](#__RefHeading___Toc317435667)

[8.2. Recruitment and consent 26](#__RefHeading___Toc317435668)

[8.3. Minors 26](#__RefHeading___Toc317435669)

[8.4. Benefits and risks assessment 26](#__RefHeading___Toc317435670)

[8.5. Compensation 27](#__RefHeading___Toc317435671)

[8.6. Incentives 27](#__RefHeading___Toc317435672)

[9. Administrative aspects and publication 28](#__RefHeading___Toc317435673)

[9.1. Handling and storage of data and documents 28](#__RefHeading___Toc317435674)

[9.2. Amendments 28](#__RefHeading___Toc317435675)

[9.3. Annual progress report 28](#__RefHeading___Toc317435676)

[9.4. End of study report 28](#__RefHeading___Toc317435677)

[9.5. Public disclosure and publication policy 28](#__RefHeading___Toc317435678)

[10. References 29](#__RefHeading___Toc317435679)

[Attachment 1: Flow Chart 32](#__RefHeading___Toc317435680)

[Attachment 2: Skin Prick test result form 33](#__RefHeading___Toc317435681)

[Attachment 3: Preparation of the DBPCFC day 35](#__RefHeading___Toc317435682)

[Attachment 4: Dosing schedule food challenge 43](#__RefHeading___Toc317435683)

[Attachment 5: Scoring system DBPCFC 44](#__RefHeading___Toc317435684)

**LIST OF ABBREVIATIONS AND RELEVANT DEFINITIONS**

| ACQ | Asthma Control Questionnaire |
| --- | --- |
| BAT | Basophil Activation Test |
| CCMO | Centrale Commissie Mensgebonden Onderzoek (Dutch competent authority) |
| CH | Case History |
| CRD | Component Resolved Diagnosis |
| DBPCFC | Double Blind Placebo Controlled Food Challenge |
| EAACI | European Academy of Allergy and Clinical Immunology |
| EC | Ethics Committee |
| ELISA | Enzyme Linked Immuno solvent Assay |
| FA | Food Allergy |
| FAQLQ-TF | Food Allergy Quality of Life- Questionnaire- Teenager Form |
| FAQLQ-PF | Food Allergy Quality of Life- Questionnaire- Parent Form |
| FAQLQ-CF | Food Allergy Quality of Life- Questionnaire- Child Form |
| FcεR | Fcε-receptor |
| HEK | Human Embryonic Kidney 293 cells |
| HEP | Histamine Equivalent Prick |
| IgE | Immuneglobuline E |
| LOAEL | Lowest Observed Allergic Event level |
| Ml | Milliliter |
| MRA | Mediator Release Assays |
| NOAEL | No Obeserved Allergic Event Level. |
| OFC | Oral Food Challenge |
| PMNs | Polymorphonuclear cell family |
| RBL | Rat Basophil Leukemia |
| SPT | Skin Prick Test |

# Summary

**Rationale:** There is an urgent need for improvement of diagnostic procedures in food allergy (FA) testing, as insufficient sensitivity and false-positivity and false-negativity hamper the currently available tests. To optimise the diagnosis of FA this study aims to improve two existing types of Mediator Release Assays (MRAs), the basophil activation test (BAT) and the rat basophil leukemia test (RBL-test). To achieve improvement of these MRAs, cashew nut allergy will serve as a model to test and validate the assays. This model is chosen because there are strong indications that cashew allergy prevalence is rapidly increasing, and is particularly affecting children.

**Objective**: The hypothesis pertinent to this proposal is that an accurate history, conventional markers of sensitization, IgE antibodies in the serum and positive SPT, and more advanced techniques (MRAs and component resolved diagnosis (CRD)) might predict clinical allergy to cashew nuts. Thereby, the aim of this study is to improve diagnostic procedures for food allergies.

**Study design:**

Interventional study. Non-drug study. Double-blind randomized food challenge. Duration per patient approx. 8 weeks. After 6 months the FAQLQ questionnaire will be completed once more.

**Study population:**

The aim is to recruit at least 200 participating children (age 2- 17 (inclusive) years) sensitized to cashew nut. Three clinics will include patients and they will carry out the clinical part of the study: Erasmus MC Rotterdam, location: Kinderhaven, Havenziekenhuis; Reinier de Graaf Gasthuis Delft, University Medical Center Groningen.

**Study intervention (if applicable):** 1 additional skin prick test, 1 additional blood sampling 12 ml, completion of questionnaire. NB: the food challenge tests are part of the current regular diagnostic procedures.

**Nature and extent of the burden and risks associated with participation, benefit and group relatedness:**

- The volunteers will visit the hospital for 4 times, 3 questionnaires will be filled in by either the parents (age 2-6 years) or the children (age 6-12), skin prick tests will be performed (total of 8) and 2 blood samples will be drawn. Furthermore the child will undergo a Double Blind Placebo Controlled Food Challenge (DBPCFC) on 2 different days.
- The burden and risks will be minimal due to extensive experience with DBPCFCs. Nevertheless, we are aware, that the outcome of the DBPCFC is unpredictable and reactions may occur in the patients. Therefore, the DBPCFC will take place at the children’s department, at the medium care unit, provided with all necessary rescue medication.
- The study can only be done in this group of children because we find high numbers of sensitization to cashew nut allergens in very young children and the DBPCFC is the only diagnostic tool we have available till now.
- The above described method is the normal diagnostic protocol (golden standard) we use to prove or reject an IgE mediated food allergy in children and adults. The only extra tests we perform for the study is are extra questionnaires, 2 extra blood samples for the Mediated Release Assay’s (MRA’s) and 6 extra skin prick tests to measure cross- reactivity with other foods.

**Possible benefit:**

The possible benefit for the patient is a clearer diagnosis concerning their possible IgE mediated cashew nut allergy and thus better dietary and lifestyle councelling.

# 1 Introduction and rationale

## 1.1. Current status of allergy diagnosis

In food allergy (FA) diagnosis, the double blind placebo controlled food challenge test (DBPCFC) is considered as the ‘golden standard’. The ‘simpler’ test types, such as *in vitro* tests based on IgE-binding (ELISA, CAP, RAST) often lead to false-positive or false-negative outcomes and do not correlate well with clinical observations, which leads to a limited reliability of their outcomes for e.g. clinical evaluations. Therefore, DBPCFC is usually not only required for initial diagnosis, but also for follow-up diagnostic procedures. DBPCFC is, however, very costly, time consuming, labour intensive, burdensome to patients and not without danger due to the chance of anaphylactic reactions. In addition, it gives no information on the responsiveness of patients to separate allergenic with different allergenic characteristics proteins in food matrices, whereas, particularly in many type of food allergens, a food contains multiple allergenic proteins.

So far, a careful side-by-side comparison of patient samples in *in vitro* assays, skin prick tests and mediator release assays has not been performed with newly emerging and potentially serious allergies, like cashew nut allergy.

Cost-effective, less-burdensome diagnostic procedures with increased relevance for clinical practice can fill an existing gap in current allergy diagnosis, and gain a considerable share in the diagnostic market for allergy.

## 1.2. Cashew nut allergy as a model to evaluate diagnostic procedures

In 1998, investigators found that 40% of 142 French peanut-allergic children were sensitized to cashew nut [1], whose intake can cause anaphylactic reactions in sensitized subjects [2-3]. At the Royal Children’s Hospital in Melbourne, in a five year period 117 anaphylactic reactions occurred in children, where peanut (18%) and cashew nut (13%) where the most common cause of anaphylaxis.[4]

There are several explanations for the occurrence of an allergic reaction at the first intake of cashew nut. First, cashew nut allergies can develop ‘in utero’ or during breast-feeding when the mother is eating cashew nuts. Secondly, cashew nut, pistachio and mango *(Anacardium* family) are botanically related *,* which causes cross-reactions. Several companies incorporate mango in food products for children from 6 months old. Finally, allergy to hazelnut might lead to allergic reactions to cashew nuts in some patients. Investigators [5]described 55% homology of allergens from hazelnut and cashew nut. It is known from literature that allergic reactions caused by cross-reactivity are less severe than reactions caused by primary sensitization [6]. It remains unclear if this is also true for sensitization to cashew nut, as most studies on cashew allergy report severe i.e. systemic reactions [3, 7].

## 1.3. Preliminary results

In Kinderhaven hospital, a multidisciplinary outpatient clinic for allergic children (department of the Erasmus MC) ,a retrospective study was performed in children with possible cashew nut allergy. In an 18 month survey we identified and tested 94 children with a suspected cashew nut allergy (mean age 7.1 years, range 15 months – 17 years) out of 290 subjects with a possible FA. Of these 94 children 69 (73%) were sensitized to cashew nut. Examining the case histories, we found a striking high number of “never eaten cashew nut” and “unknown” (n = 46; 65%) cases of cashew nut allergy. In 6/69 (9%) children, anaphylactic reactions occurred after eating cashew nuts. Furthermore, 17/69 (24%) reported oral allergy symptoms (OAS) and/or angio-edema (AO). In 26/ 69 cases with positive sensitization to cashew nut, the parents reported possible symptoms caused by pistachio nuts. Skin test with pistachio nut were positive in 24/ 26 of these cases. Co-sensitization was seen with hazelnut: 80% and peanut: 65%. This indicates the presence of possible cross reactivity between different nut allergens. Only 4 children were mono-sensitized to cashew nut.

In summary, from the literature and preliminary results it can be seen that sensitivity to cashew nuts is highly prevalent, representing a mixed population in terms of disease severity. Cross-reactivity can be a severity modifying factor. The preliminary results indicate that these patients are frequently seen. With this background cashew nut allergy provides an attractive model to validate diagnostic tools.

# 2. Optimization of the diagnosis of cashew nut allergy

## 2.1. Components of diagnostic procedures

Mediator Release Assays (MRAs) may circumvent, in particular in the setting of food allergies, the drawbacks of on the one hand IgE-based assays (‘overly’ simple leading to an unacceptably high level of erroneous outcomes), Skin Prick Test (SPT) (confounding cross-reactions, instability of allergens, thus erroneous outcomes) and on the other hand DBPCFC (expensive, burdensome, no resolution at level of allergenic protein, potentially not without risk):

- In MRAs, allergen cross-linking of IgE (allergen binds to two distinctive IgE’s) that is bound to effector cells is required to achieve a positive response. This strongly enhances relevance of such tests in comparison to tests that are based on a single IgE-binding only (as seen in ELISA), as only biologically functional IgE and allergens are detected, and thus potentially discriminates between patients that are only sensitized and patients that have a clinically relevant allergy
- Only a, relatively small, blood sample from test persons is required, thereby avoiding ingestion of food in DBPCFC and the associated risks of strong reactions. Interference of components from skin, as may occur in SPT, is avoided
- This test can provide physiologically relevant information to the level of individual allergenic proteins. This information is relevant for the clinical interpretations, but can also serve to develop physiologically relevant methods to evaluate the allergenic potential of nutritional products, thus creating safer food products for consumers
- The MRA can be utilized as a safe and easy method to establish threshold levels for clinical relevant allergen content, needed to define the “may contain labelling” directive of food allergens in processed nutrition, incurring no risk for patients.
- The MRA-assays are much more cost-effective than DBPCFCs.

Basically, two types of MRAs exist, both of which will be included in this research program:

The basophil activation test (BAT) is performed with patient’s own IgE-loaded basophilic cells, which are subsequently exposed to functional, cross-linking-capable, allergen preparations. The rat basophil leukemia test (RBL) makes use of a (rat-derived) cell line that expresses the human Fcε-receptor (FcεR). These cells can be ‘loaded’ with IgE from a patient’s serum, an operation that can be done in a stage at which a certain number of sera (that can stably be stored for long intervals) can be analysed simultaneously. RBL-cells can be used as a ‘vehicle’ to analyse any type of food allergy, provided suitable allergen preparations are available.

The BAT is hampered by operational logistics being a cost-increasing factor. The RBL-test has limited sensitivity. Both aspects will be addressed in this project.

Currently, it is not known to what extent the outcome of an oral challenge with cashew nuts can be predicted by less invasive procedures. This project will present an unique opportunity to investigate the potential of less invasive allergy diagnostic tests, in particular cell-based assays, and validate these tests against the clinical background. A new approach of diagnosing food allergy is the use of component resolved diagnosis (CRD), which comprises the determination of IgE by ELISA that recognises isolated allergenic proteins, which are either produced by recombinant expression of allergen-encoding cDNAs or by purification from natural allergenic foods.

However, such CRD can also be performed, and be more relevant, with MRAs such as rat basophil leukemia cells (RBL) [8] or basophile activation tests (BAT) [9-10] which analyse the cross-linking ability, and thus the functionality, of allergens. This circumvents potential false-positive outcomes which may result from IgE-binding to single epitopes without the necessity to provoke cross-linking to create a response in the assay.

From a clinical point of view but also to evaluate the importance of MRAs, there is a need to estimate how accurately different components of the FA diagnosis (i.e. history, conventional markers of sensitization and more advanced techniques (cell-assays and CRD) in [11] can predict clinical allergy to cashew nuts as measured by DBPCFC.

Assuming that MRAs will be important in the diagnosis of FA, the question should be answered to what extent an accurate diagnosis of FA decreases the burden of disease. For that reason food related quality of life will be measured with instruments developed by the UMCG [12]. If a definite diagnosis has a beneficial impact on quality of life, this observation will underwrite the importance of using non-invasive diagnostic procedures (i.e. MRAs).

## 2.2. Cross reactivity between cashew nuts and other allergens

As primary and secondary sensitizing allergens may have a differential effect on the severity of allergic reactions, further research to cross reactive cashew nut allergens is warranted.

To date 3 major allergenic proteins have been identified in cashew nut and these are available as recombinant proteins. [13-14] Ana o 1, one of these allergens, is a vicilin (a 7S-seed storage protein). Previous research failed to show a significant cross-reactivity of Ana o 1 with the 7S-storage protein in peanut (Ara h 1), in hazelnut (Cor a 11), and walnut (Jug r 2) [15]. In addition Ana o 2 (a 11S globulin) and Ana o 3 (2S Albumin) have been identified, which are seed storage proteins as well. Recently, several 11S globulins with reactivity to patient IgE were found [16-17].

Although the major allergenic proteins are identified, cross reactivity between recombinant allergens from cashew nut and the allergens from pistachio nut and mango (*Anacardium* family) is hardly examined [18]. Pallawid Tawde et al. [5] found that the identified N-terminal amino acid sequences of cashew 11S globulin (*Ana o 2*) possessed significant homology (45-58% identity and 63-74% similarity) to the 11S globulins from diverse tree nuts, seeds, and legumes. These findings contribute to the hypothesis that hazelnut might be the primary sensitizer in some cases. It is known from literature that allergic reactions caused by cross-reactivity are less severe than reactions caused by primary sensitization.

MRAs will offer improved possibilities to analyse the molecular mechanisms underlying these cross-reactive phenomena and their physiological relevance. This will contribute to improved clinical diagnosis and improved patient information, without the need to involve clinical test procedures for this.

# 3. Study objectives

## 3.1. Primary objectives

The hypothesis pertinent to this proposal is that an accurate history, conventional markers of sensitization, IgE antibodies in the serum and positive SPT, and more advanced techniques (MRAs and component resolved diagnosis, CRD) might predict clinical allergy to cashew nuts. However, the diagnostic value of these predictors has to be determined by DBPCFCs, which is the gold standard for the diagnosis of food allergy. Thereby, the aim of this study is to improve diagnostic procedures for food allergies. The following research lines are therefore implicated:

- *Optimization of the diagnosis of cashew nut allergy; development of new* diagnostic tools

Improved Mediator Release Assays (MRAs) will be developed e.g. basophil activation test (BAT) and rat basophil leukaemia test (RBL). The researchers aim to optimize the diagnosis of nut allergy, by using the different components: 1) history, 2) conventional markers of sensitization, 3) MRA’s and 4) CRD i.e. IgE-levels of Ana o 1, Ana o 2 and Ana o 3, to finally predict the outcome of the DBPCFC. Measure the effect of improved diagnostic procedures on quality of life.

- *Develop a diagnostic model with new Mediator Release assays to predict the outcome of the DBPCFC*

Predictive modelling and regression techniques will be used to accomplish and identify the optimal set of diagnostic procedures for the diagnosis of cashew nut allergy. In fact, these models might be useful for other nuts as well. In that way it will accomplish a breakthrough in the diagnosis of food allergy.

## 3.2. Secondary objective

- *Cross reactivity between cashew nuts and other allergens*

The question will be addressed as to whether cashew nut is the primary sensitizing agent in cashew nut allergy. For that purpose a profile of co-sensitizing allergens (e.g. peanut, other nuts, and mango) needs to be accomplished. Because most reported cashew allergies are severe, the mechanisms of cashew nut and cross reactivity will be studied in more detail.

# 4. Investigational plan

## 4.1. Study design

Interventional study. Non-drug study. Double-blind randomized food challenge. Duration per patient approx. 8 weeks. After 6 months the FAQLQ questionnaire will be completed once more.

The aim is to recruit at least 200 participating children (age 2- 17) sensitized to cashew nut. In this study this number should be sufficient to measure prevalence, investigate cross-reactivity, develop and validate MRAs, and to build models identifying a set of predictors up to 10 for the outcome of DBPCFC’s. This assumes, based on our elaborative experience in similar studies, that 1600 children are needed to obtain 200 sensitized children undergoing a DBPCFC (see flow chart, Attachment 1). Based on recruitment from 3 medical centres this should be realistically feasible in 36 months.

Questionnaires need to be filled in by the participants in cooperation with their parents, concerning a possible food allergy, and actual diet. A blood sample will be taken. Additional skin prick tests will be performed with birch pollen, hazelnut, pistachio nuts, peanuts, and mango. The participating children will undergo a DBPCFC with cashew. Selected experiments in blood will be performed to study the mechanism of primary sensitization and potential cross-reactivity, e.g. by ELISA, immunoblot inhibition experiments, BAT, RBL, and the use of recombinant allergens, like Ana o 1, Ana o 2, Ana o 3.

We anticipate to improve the sensitivity of the RBL-2H3-assay by genetically increasing the expression of the FcεR type I receptor via the use of different expression plasmids and by transfecting more suitable cell lines (human HEK293 cells with which we have ample experience) with this receptor under control of a strong constitutive promoter.

In addition, the BAT assays will be modified by using optimized conditions for the patient-derived basophiles, the determination of the *in vivo* IgE loading of the cells, and the preparation of the allergens or fractions thereof for stimulation. The consortium has ample experience in handling these cells.

In total the children will visit the hospital for 4 sessions after the first visit. In one session the skin prick test, questionnaires and blood samples will be taken, in the following 2 sessions DBPCFC’s will take place. In a final visit the patient (parents) will be informed whether or not a clinical relevant cashew nut allergy is confirmed and advise will be given concerning the diet in case the DBPCFC is positive. In case the challenge is negative an open challenge will be performed to confirm the absence of cashew nut allergy. For skin tests in house manufactured extracts [19] will be prepared and distributed by the Erasmus MC. Skin prick test results will be scanned and index calculations will be made.

## 4.2. Existing infrastructure

The scientific challenges require a multidisciplinary approach and the input of two PhD students with different skills. Expertise is required on the following fields: allergen purification and characterization, cell culture of human cells, different cell lines and knowledge of cellular transfection and basic molecular biological techniques (like cloning and expression analysis by real time quantitative PCR), and *in vitro* IgE analysis, skin prick testing and DBPCFC, recognition and interpretation of clinical disease expression. This task is enormous and can be accomplished only by intensive collaboration between centres of excellence in this field. Three clinics, experienced in the field, will include patients and will carry out the clinical part of the study, whereas *in vitro*-analysis will be carried out in one laboratory.

Three clinics will perform the clinical part of the study:

- Erasmus MC Rotterdam, location: Kinderhaven Havenziekenhuis, food challenge in Sophia Kinderziekenhuis
- Reinier de Graaf Gasthuis Delft
- University Medical Center Groningen.

Immunoblot experiments, recombinant allergens, BAT and RBL assays:

- Wageningen University.

Prognostic modelling and the use of regression techniques:

- Erasmus MC Rotterdam, Centre for Medical Decision Making.

# 5. Population

All children (2 to 17 years) visiting the outpatient department of the participating clinical centres will be screened for sensitization for cashew nut by skin prick test and/or determination of sIgE to cashew nut.(CAP-FEIA, ALK- Abello)

## 5.1. Inclusion criteria

- Age: 2-17 year.
- Children with a positive skin prick test (HEP > 0.21) and/ or detectible sIgE (>0.35) to cashew nut.
- History of positive reaction to cashew nut or an unknown reaction (because of never ingested).
- Written informed consent parents and child (≥ 12 year old).

## 5.2. Exclusion criteria

- History of severe or uncontrolled asthma (investigator’s opinion).
- Severe eczema defined as TIS (Three Item Severity) eczema score (> 6).
- Immunological diseases, cardiovascular diseases or malignity.
- Severe psychosocial problems.
- Not able to stop anti-histamine medication for a short period.
- Use of beta-blockers.
- The patient is allergic to one or more of the ingredients of the food matrix, unless a suitable substitute for the ingredient in question can be found.
- Unable to speak and understand the Dutch language properly.
- Not willing to comply with the study procedures.

## 5.3. Sample size calculation

The aim is to recruit 200 children (age 2-17). In the three year period, all children who are sensitised to cashew nuts enter the study. We expect that 200 children will enter the study. Since the proportion of children that will have a clinical allergy to cashew nuts is unknown, we assume that 50% of the sensitised children experience a clinical allergy. With approximately 100 cases (50% of 200), a reliable prediction model can be constructed when we consider not more than 10 candidate predictor variables, using the 1:10 rule as a guidance [43,44].

This assumes, based on our elaborative experience in similar studies, that 1600 children are needed to obtain 200 sensitized children undergoing a DBPCFC. Which means that: 10 refuse extra skin test, 77% is not sensitized, 25% refuse BDPCFC and 20 is not eligible for analysis. (flow chart; Attachment 1).

Based on recruitment from 3 different medical centres this seems feasible in 36 months.

# 6. Methods

## 6.1. Visit schedule and assessments

Patient who meet all the criteria will be asked to participate in the study by the investigator during the normal visit to the physician. The patient/ parents will be given the Patient information form (PIF). After two weeks the patients/ parents will be approached by telephone by the investigator whether or not they are willing to take part in the study. If positive the patient will be invited for the first visit. For this study the patient/parents will come to the hospital for 3 sessions. Inclusion will be stopped when a total of 200 children is included in the three clinics.

## 6.2. Patient numbering

Every patient is uniquely identified by a combination of his/her centre number (Erasmus MC nr 111; Reinier de Graaf Gasthuis nr 222; University Medical Centre Groningen nr 333), and patient number.

In each centre, the first patient is assigned patient number 1, and subsequent patients are assigned consecutive numbers. All results will be collected in the Case Report Form (CRF).

## 6.3. Data to be collected/assessments

### 6.3.1. History

### Patient demographics/other baseline characteristics

Patient demographics and baseline characteristics will be collected in the CRF at Visit 1.

Data collected will include date of birth, sex, race, ethnicity, height and weight, relevant medical history and current medical conditions, prior concomitant medications.

A questionnaire (CRF) will be used to score on allergic complaints. Inhalation and food allergy complaints will be scored in the CRF, with time of onset of complaints.

**6.3.2.Dietary history on consumption of cashew nut**

Additionally, the dietary history will be entered in the CRF. In this way patients are allocated to one of the following categories [20], according to the ingestion of cashew nut:

- Only skin contact, no reaction
- Only skin contact, allergic reaction
- Mucosal contact only, no reaction
- Mucosal contact only, allergic reaction
- Cashew nut present in diet , no reaction:
  - when incorporated in other foods
  - < ¼ cashew nut (< 175 mg cashew nut) : < 39 mg E
  - ¼ - ¾ cashew nut (175 mg – 525 mg): 39 - 111 mg E
  - > ¾ cashew nut (> 525 mg cashewnoot) : > 117 mg E
  - on a regular basis
  - unknown
- Cashew nut was present in diet, most severe allergic reaction:
  - when incorporated in other foods
  - < ¼ cashew nut (< 175 mg cashew nut) : < 39 mg E
  - ¼ - ¾ cashew nut (175 mg – 525 mg): 39 - 111 mg E
  - > ¾ cashew nut (> 525 mg cashew nut) : > 117 mg E
  - On a regular base
  - Unknown
- Presence of cashew nut is unknown
- Cashew nut is not present

### 6.3.3. FAQLQ Questionnaire

Food Allergy Quality of Life Questionnaire – Child form 6-12 years (FAQLQ-CF). 12-18 years (FAQLQ-TF) and Parent form 2-6 years (FAQLQ-PF) [12, 21-22]

The FAQLQ is a disease specific instruments that evaluate the impact of food allergy on patients’ health-related quality of life along 4 domains: allergen avoidance and dietary restrictions, emotional impact, risk of accidental exposure, and food allergy

related health. The FAQLQ is a 29-item questionnaire, self-administered and will be completed by the patient at the clinic at screening (visit 1), and 6 months after the DBPCFC. The hypothesis is, that patient QOL is importantly increasing after a clear cut diagnosis of food allergy. Thus, patients will have time to get used to the new diagnosis and consequently, the Questionnaires will be repeated a half year after the DBPCFC’s [23].

The total FAQLQ score is the mean score of all items with a range of 1 ‘no impairment’ to 7 ‘maximal impairment’. The total FAQLQ score is calculated by adding up the scores and dividing by the number of answered questions at each time point. Within diagnostic evaluation of the cashew nut allergy comparison will be performed for the change from the screening total score to the end of the 5^th^ visit. The statistical significance and 95% confidence intervals will be calculated using paired t-test. For each treatment arm, estimated mean change from baseline with associated 95% confidence interval and p-value will be displayed.

### 6.3.4. Physical examination

A physical examination will be performed during visit 1.

## 6.4. Time table

| **Visit 1**  **week 1** | **Visit 2 week 4** | **Visit 3**  **week 6** | **Telephone**  **Week 7** | **Final visit**  **Week 8** |
| --- | --- | --- | --- | --- |
| Written informed consent  Medical history  Physical examination  Skin prick tests  Blood samples (12 ml)  FAQLQ questionnaire(s) *) | DBPCFC  Session 1 | DBPCFC  Session 2 | **Result DBPCFC**  When positive | Dietary advice |
|  |  |  | When negative | Advice introduction cashew nut at home |
| *) Patients will be asked to fill in the FAQL Questionnaire at the first visit and the second time will be a half year after the DBPCFC. This second Questionnaire will be filled in at home, so the patients will receive the Questionnaire at home with an answering envelope. | | | | |

## 6.5. Skin prick tests

Skin prick tests with 2 positive controls, one negative control, birch pollen, cashew nut pistachio, mango and peanut will be carried out according to international guidelines and performed by a dedicated allergy nurse. Fifteen minutes after performing the skin tests, the contours of the reaction will be encircled with a fine-tip pen and transferred to a record sheet using transparent tape.(“Skin Prick test result form”, Attachment 2) Mean weal of the surface within the encircled area, the Histamine Equivalent Prick index (HEP-index) will be calculated. This will be done by dividing the area of the SPT of the allergen by the area of the mean of two histamines of the SPT using a scanning device (Hewlett Packard 2400c, Houston, TX, USA) and software that was earlier developed in the Erasmus MC hospital [www.PAAMOST.nl]. This method showed higher accuracy and reproducibility than the manually determined mean weal diameter [24].The cut-off value of 0.21 is used when interpreting the HEP-index. This corresponds to a weal size of 3mm, the internationally accepted cut-off level for SPT for the mean weal diameter. This classification is a modification of the grading system described by Niemeijer et al. [25].

## 6.6. Double blind placebo controlled food challenge (visit 2 and 3)

### 6.6.1. Background

Guidelines recommend using oral food challenges for diagnosing Food Allergy (FA). While a single-blind or an open-food challenge may be considered diagnostic under certain circumstances the double blind placebo controlled food challenge (DBPCFC) is the gold standard approach [26]. Investigators agree that verification of clinical reactivity requires well-designed oral food challenge testing [27]. Because of the inherent risk of serious reactions, an oral food challenge must be conducted at a medical facility with medical supervision and appropriate medication and devices on hand. The challenge test is carried out while the patient is on minimal or no symptomatic medication. The test should be designed and performed under medical supervision primarily for the patient’s safety, but also to document the dose that provokes the reaction and to administer symptomatic treatment, which may require management of anaphylaxis. The medical personnel should have experience in carrying out such challenges. Ideally the oral food challenge begins with a low dose (intended to be lower than a dose that can induce a reaction [28]. While monitoring for any allergic symptoms, the dose is gradually increased, until a cumulative dose at least equivalent to a standard portion for age is consumed. Because of the risk of a severe reaction, intentional challenge should be avoided in patients who have recently experienced a life-threatening reaction to cashew nut, particularly if it occurred more than once and if the patient is sensitised for cashew nut. Studies differ in starting doses, typically in the range of the quantity of the food for the most sensitive subjects to produce an objective, but mild reaction. Studies also differ in challenge procedures, the form of the food used, the matrix in which the allergen was presented and the weight accorded to subjective and objective manifestations. Though there is currently no internationally accepted, standardized protocol for performing and interpreting DBPCFCs, several Oral Food Challenge Protocols have been published. Efforts to standardize OFC began in 2004 with the position paper from the European Academy of Allergology and Clinical Immunology [29] and more recently the Adverse Reactions to Food Committee of the American Academy of Allergy, Asthma & Immunology published a review of Oral Food Challenge tests [30].

In a recent study [42], the results of double blind, placebo controlled food challenges with cashew nut were examined in order to generate threshold distribution curves for cashew. This study included the results from 31 challenges with cashew which resulted on positive allergic reactions. Children were included in the test regardless of the severity of the reaction to cashew by history. Children and/or their parents had to consent to the test and the children had to be medically stable at the time of the test. The recipe for these challenges includes a starting dose of 1,75 mg cashew protein (corresponds to 12 mg of cashew) with a final total dose of 4 grams of cashew, divided over 6 incremental doses given at 30 minute intervals. There were no life-threatening reactions and no patients required epinephrine administration or in patient care.

### 6.6.2. Patients

Preparation:

In case the patient presents with a concurrent illness, e.g. common cold, the DBPCFC should be postponed until the patient has recovered. Before the DBPCFC, the patient may consume his/ her normal breakfast.

On the dag of the Challenge a standardized Questionnaire needs to be filled in by the physician whether challenge may be performed. (“Starting Form DBPCFC day”, Attachment 3).

The patient is scheduled for a DBPCFC with cashew nut in one of the participating centres of the study. The patients is to remain to stay on a cashew nut free diet prior to the challenge, if indicated (only when clinical symptoms to cashew nut are suspected).The challenge is performed no longer than 6 months after the skin prick tests and the taking of blood samples. Patients must be in good health, and any concomitant allergic diseases e.g. asthma, allergic rhinitis, or atopic dermatitis should be under optimal control.

In case the patient presents with a concurrent illness, e.g. common cold, the OFC should be postponed until the patient has recovered. Before the OFC, the patient may consume his/ her normal breakfast.

### 6.6.3. Performance

The personnel involved in the food challenge procedure must be trained in the management of

acute allergic reactions and equipment for resuscitation (including adrenaline for injection and oxygen) must be readily available. A crash cart must be readily available on site during all food challenges and study drug administrations.

Clinical symptoms and overall condition have to be stable and antihistamines have to be stopped at least 72 hours prior to the test, if possible. Weight and height of the child are measured and the exact doses of the drugs which might be necessary (rescue medication: adrenaline auto-injector (e.g. Epipen), adrenaline, clemastine, other antihistamines, xylometazoline and salbutamol, short acting bronchodilator) are computed and prepared:

**Oral**

Medication Age Concentration Dose Note

| Fenistil oral solution |  | 1 drop/ kilogram | ... drops |  |
| --- | --- | --- | --- | --- |
| Xyzal oral solution | 2-3 year | 0.5 mg/ml | 1.25mg = 2.5 ml |  |
| Xyzal oral solution | 4-6 year | 0.5 mg/ml | 2.50 mg = 5 ml |  |
| Aerius melt tablet | 4-6 year |  | 2.5mg |  |
| Xyzal tablet | ≥ 6 year |  | 5 mg |  |
| Aerius melt tablet | ≥ 6 year |  | 5mg |  |
| Semprex capsule | ≥ 12 year |  | 8 mg |  |
| Prednisone |  |  | 1-2mg/kg | Max 40 mg |

**Inhaled**

**Medicat**ion Age Concentration Dose Note

| Xylometazoline | 2-6 year | 0.05%-solution | 1-2 drops or 1 spray/nostril | Max 3x/day |
| --- | --- | --- | --- | --- |
|  | >6 year | 0.1%-solution | 2-3 drops or 1 spray/nostril | Max 6x/day |
| Salbutamol | <4 year | 2.5mg/2.5 ml | 2.5 mg | Nebulize |
|  | >4 year | 5mg/2.5 ml | 5 mg |  |
| Atrovent | <4 year | 500mcg/ml | 250 mcg= 1ml | Nebulize |
|  | >4 year | 500mcg/ml | 500 mcg= 2ml |  |

**Parenteral**

Medication Weight Concentration Dose i.m./i.v. Note

| Adrenaline |  | 1mg/ml | 0.01mg/kg = .... ml | i.m. |  |
| --- | --- | --- | --- | --- | --- |
| Epipen junior | < 30 kg |  | 0.15 mg | i.m. |  |
| Epipen | > 30 kg |  | 0.30 mg | i.m. |  |
| Tavagyl |  | 1mg/ml | 0.025mg/kg = .... ml | i.m./i.v. |  |
| Hydrocortisone |  | 50mg/ml | 10mg/kg = .... ml | i.v. | Max 200 mg Application: slow |
| Dexamethasone |  |  | 0.1mg/kg = .... mg | i.m./i.v. | Max 8mg |

### 6.6.4. Randomization, blinding and preparation

The active test food recipe and placebo recipe for cashew nut are provided monthly, deep frozen by the (NIZO Processing centre; Ede, the Netherlands). The verification code and the preparation of the recipe (ingredients used) are verified by a second individual. A randomization list will be prepared centrally for each participating centre. In each centre an unblinded nurse will be responsible for the preparation of the challenge material in accordance with the randomization list.

The placebo and active test food challenges are administrated on separate days with at least two weeks interval in between. The child is tolerant to the ingredients of the food matrix, as ascertained for by the dietician. A validated recipe “cashew nut in gingerbread” is used as challenge material [31]. Cashew nuts will be provided by Intersnack; Doetinchem. The challenge procedure includes an eight-step incremental design in which progressively greater quantities of the same allergenic food are administrated. The interval between the challenge doses is 30 minutes.[32]

The starting dose will be 1 mg of cashew protein equivalent. As the LOAEL (lowest observable adverse effect level) of peanut appears to be 3 mg of peanut protein [33], we decided to choose 1 mg of cashew. We will use an incremental schedule of 3 fold increase by which 1, 3, 10, 30, 100, 300, 1000 and 3000 mg of cashew protein (or placebo) will be administered. (“Challenge doses Cashew DBPCFC”, Attachment 4)

In a recent study [34], the results of double-blind, placebo-controlled food challenges with cashew were examined in order to generate *threshold distribution curves* for cashew. This study included the results from 31 challenges with cashew which resulted on positive allergic reactions. Children were included in the test regardless of the severity of the reaction to cashew by history. Children and/or their parents had to consent to the test and the children had to be medically stable at the time of the test. The recipe used for these challenges use a starting dose of 1,75 mg cashew protein (corresponds to 12 mg of cashew) with a final total dose of 4 grams of cashew, divided over 6 incremental doses given at 30 minute intervals. There were no life-threatening reactions and no patients required epinephrine administration or in patient care.

Schedules using semi-logarithmic increases are associated with good safety and are more often used in other centres. [35]. Smaller increments (e.g. doubling) may further enhance safety but would also significantly increase the time necessary to complete the challenge procedure. Therefore, the proposed scheme is expected to represent a suitable challenge regimen, balancing safety and feasibility.

| Dose | Protein (mg) | Protein cum (mg) | Calculation | Administered amount of food (g) | Administered amount of food cum (g) |
| --- | --- | --- | --- | --- | --- |
| 1 | 1 | 1 | (120*/3180) x 1 | 0,04 | 0,04 |
| 2 | 3 | 4 | (120*/3180) x 3 | 0,11 | 0,15 |
| 3 | 10 | 14 | (120*/3180) x 10 | 0,38 | 0,53 |
| 4 | 30 | 44 | (120*/3180) x 30 | 1,13 | 1,66 |
| 5 | 100 | 144 | (120*/3180) x 100 | 3,77 | 5,43 |
| 6 | 300 | 444 | (120*/3180) x 300 | 11,32 | 16,75 |
| 7 | 1000 | 1444 | (120*/3180) x 1000 | 37,74 | 1 – 4 years 54,49 |
| 8 | 1736 | 3180 | (120*/3180) x 1736 | remaining 65,51 | ≥ 4 years 120,00 |

For children of 4 years and above, a total amount of 120 grams of test food (containing 3180 mg protein or 15 grams of cashew nut or ~22 cashew nuts) will be administered.

For children younger than 4 years of age, this will be half of the total amount, i.e. 60 grams of test food (containing 1444 mg protein or 7,5 grams of cashew nut or ~11 cashew nuts).

On both placebo and active test food challenge, a total of 120 grams of test food is administered.

### 6.6.5. Assessment protocol for the outcome

Challenge sessions in which children consume less than 75% of the maximum challenge dose are considered invalid. The challenge is discontinued when objective allergic symptoms occur, or subjective allergic symptoms occur twice on two successive administrations of the challenge material [28].

Judgments about proceeding must balance safety against the certainty of the challenge outcome. Objective symptoms and signs are defined as (angio) edema, urticaria, exacerbation of atopic eczema, vomiting, diarrhoea, lip or tongue swelling, rhino-conjunctivitis, stridor, coughing, wheezing, hoarseness, collapse, tachycardia and hypotension.

Subjective symptoms are defined as exacerbation of generalized itch (in case of atopic eczema), abdominal pain, nausea and/or cramp, oral allergy symptoms, itchy throat or sensation of throat swelling, difficulty in swallowing and other symptoms such as drowsiness and irritability. Immediate symptoms are defined as symptoms occurring during the challenge or within two hours after the last challenge dose.

A standardized scoring system will be used to score objective and subjective symptoms in the patients. (“Scoring system DBPCFC Cashew study” Attachment 5)

Symptoms

| **Objective** | **Subjective** |
| --- | --- |
| **Skin** | **Skin** |
| Urticaria and angioedema | Itching |
| Redness of the skin |  |
| Exacerbation of atopic eczema |  |
|  |  |
| **Respiratory** | **Respiratory** |
| Rhinitis | Feeling of dyspnea |
| Wheezing |  |
| Coughing |  |
| Stridor |  |
| Hoarseness |  |
|  |  |
| **Eye symptoms** | **Eye symptoms** |
| Conjunctivitis | Itching |
|  |  |
| **Gastro-intestinal** | **Gastro-intestinal** |
| Vomiting | Cramps |
| Diarrhoea | Stomach ache |
|  | Oral allergy symptoms |
|  | Itching throat |
|  | Sensation of throat swelling |
|  | Dysphagia |
|  |  |
| **Cardiovascular** | **Cardiovascular** |
| Hypotension | Weakness |
| Tachycadia | Dizziness |
| Collaps |  |
| Lose consciousness |  |
| Anafylaxis |  |
|  |  |
| **Other** | **Other** |
| Extreme crying | Psychological |
| Change of behaviour |  |

Two hours after the last dose, or when all symptoms have disappeared and the patient is in a stable condition, the patient is sent home. The patient is requested to record late onset symptoms on a scoring sheet (“Scoring system 48 hours after DBPCFC”, Attachment 6) and to contact the hospital I n case of severe symptoms. Two days after the challenge session late onset reactions are recorded by telephone using the same Questionnaire. Late onset symptoms are defined as symptoms occurring between two and 48 hours after the last challenge dose [29]. Forty-eight hours after the second challenge session, the code is broken and the outcome of the DBPCFC is assessed as follows:

| **Active food challenge** | **Placebo** | **Assessment of DBPCFC** |
| --- | --- | --- |
| Positive (or clearly more positive/ more objective than placebo) | Negative | Positive |
| Positive | Positive | Negative |
| Questionable | Questionable | Negative |
| Negative | Negative | Negative |
| Negative (or positive, but clearly less positive than placebo) | Positive | Negative |

In exceptional cases, a challenge session or total DBPCFC may be assessed as inconclusive, e.g. in case of mild symptoms at the last dose. Inconclusive DBPCFCs are repeated within 6 months.

### 6.6.6. Results, telephone contact

Patient will be informed about the result of the DBPCFC by telephone by the investigators. Appointment for the last visit will be made. During a final visit the patient (parents) will be informed by the investigator whether or not a clinical relevant cashew nut allergy is established and the diagnosis cashew nut allergy is confirmed. When a cashew nut allergy is confirmed, the patient (parents) will be advised to continue the cashew nut free diet. When the patient has no cashew nut allergy, the patient will be advised how to reintroduce the cashew nut at home [36].

## 6.7. Blood collection

### 6.7.1.IgE

For IgE measurement 1 vial of whole blood (8ml) will be drawn, centrifuged (2000 rpm, 10 min.) Serum will be stored at -20 C for further examination. Specific IgE will be measured against: Cashew allergens: Ana o 1, Ana 0 2 and Ana o 3, and to possibly cross-reacting allergens from mango and from pistachio. Rest of the serum will be stored at -20 C for the use in RBL analysis.

### 6.7.2.BAT

For the basophil activation test (BAT), 1 vials of EDTA blood (4ml) will be drawn at the first visit and sent to Wageningen immediately. From these blood samples, plasma and granulocytes are isolated via Ficoll-centrifugation. Plasma samples will be stored at -20 °C. Polymorphonuclear cell family (PMNs) will be used to improve the BAT-test to facilitate clinical application in allergy diagnosis.

### 6.7.3. RBL

For RBL analysis rest of the serum which is stored at -20 C will be used to study how these RBL-tests, or tests making use of Human Embryonic Kidney 293 (HEK) cells, can be improved to increase their sensitivity and application for clinical testing.

## 6.8. Withdrawal of individual subjects

Subjects can leave the study at any time for any reason if they wish to do so without any consequences. The investigator can decide to withdraw a subject from the study for urgent medical reasons.

# 7. Data collection and statistical analysis

We hypothesize that MRAs are superior to other tests in predicting clinically important allergy to cashew nuts. Superiority will justify their use and may lead to displacement of other diagnostic procedures. To this purpose, predictive modelling and regression techniques will be used to accomplish and identify the optimal set of diagnostic procedures for the diagnosis of cashew nut allergy. In fact, these models might be useful for other nuts as well. In that way it would accomplish a breakthrough in the diagnosis of food allergy.

We will use logistic regression analysis to study the association between candidate predictors (diagnostic tests) and cashew nut allergy. The shape of associations between continuous test results and cashew nut allergy will be studied with restricted cubic splines [37] with three knots (two degrees of freedom). If the association is non-linear, an appropriate transformation will be selected, such as the natural logarithm, square, and square root [38].

The regression coefficients in the final model will be multiplied with a shrinkage factor that is estimated with bootstrapping. Shrinkage is applied to obtain accurate predictions for new patients; without shrinkage, predictions are in general too extreme resulting in low predictions being too low and high predictions too high.

The final model with the shrunken regression coefficients will be presented in an easy to use format to facilitate clinical application, for instance a score chart or monogram.

This analytical strategy to develop a diagnostic model is according to the current state of the art [39] The strategy aims to develop a diagnostic model that can provide accurate predictions for future patients rather than predictions that are correct for the patients of the development dataset. Therefore, the selection of predictors in the model is based on multivariable modelling only, with a backward selection procedure and liberal p value (0.157 in this study). The use of higher p-values, results in inclusion of relatively weaker predictors, in the model, on the cost of possible selection of a nuisance variable. Such model performs well in new [40-41].

# 8. Ethical considerations

## 8.1. Regulation statement

The study will be conducted in accordance with the principles of the current version of the Declaration of Helsinki and in accordance with the Medical Research Involving Human Subjects Act (WMO).

## 8.2. Recruitment and consent

Patients will be recruited from the patient population of the participating clinical sites. The interest of parents and children for participation will be checked by the treating physician. In case of potential interest the children and parents will be referred to the study staff for the formal informed consent procedure. In case the treating physician is also the investigator, the informed consent procedure will be handled by another member of the study staff. An independent physician will be available.

An information letter will be available for the parents and for the children ≥12 years of age. For participation of a child a signed consent form signed by both parents (or legal guardian) is mandatory. If a child is 12 years or above, the child must give written consent as well.

Sufficient time will be allowed to consider participation, with a maximum of 2 weeks. This may be extended if deemed justified by the person handling the informed consent procedure.

## 8.3. Minors

In case the minor patient does not cooperate or shows signs of resistance, the investigator should discontinue the study immediately. The guideline of the Dutch Society for Pediatric Medicine (Nederlandse Vereniging voor Kindergeneeskunde (NVK)) for the protection of minor research subjects will be observed.

## Benefits and risks assessment

Burden/risks:

- The volunteers will visit the hospital for 4 times, 3 questionnaires will be filled in by either the parents (age 2-6 years) or the children (age 6-12), skin prick tests will be performed (total of 8) and 2 blood samples will be drawn. Furthermore the child will undergo a Double Blind Placebo Controlled Food Challenge (DBPCFC) on 2 different days.
- The burden and risks will be minimal due to extensive experience with DBPCFCs. Nevertheless, we are aware, that the outcome of the DBPCFC is unpredictable and reactions may occur in the patients. Therefore, the DBPCFC will take place at the children’s department, at the medium care unit, provided with all necessary rescue medication.
- The study can only be done in this group of children because we find high numbers of sensitization to cashew nut allergens in very young children and the DBPCFC is the only diagnostic tool we have available till now.
- The above described method is the normal diagnostic protocol (golden standard) we use to prove or reject an IgE mediated food allergy in children and adults. The only extra tests we perform for the study is are extra questionnaires, 2 extra blood samples for the Mediated Release Assay’s (MRA’s) and 6 extra skin prick tests to measure cross- reactivity with other foods.

Possible benefit:

The possible benefit for the patient is a clearer diagnosis concerning the possible IgE mediated cashew nut allergy.

## Compensation

A liability insurance which is in accordance with article 7, subsection 6 of the WMO and an insurance which is in accordance with the legal requirements in the Netherlands (Article 7 WMO and the Measure regarding Compulsory Insurance for Clinical Research in Humans of 23th June 2003) has been arranged. The latter insurance provides cover for damage to research subjects through injury or death caused by the study:

1. € 450.000,-- (i.e. four hundred and fifty thousand Euro) for death or injury for each subject who participates in the Research;
2. € 3.500.000,-- (i.e. three million five hundred thousand Euro) for death or injury for all subjects who participate in the Research;
3. € 5.000.000,-- (i.e. five million Euro) for the total damage incurred by the organisation for all damage disclosed by scientific research for the Sponsor as ‘verrichter’ in the meaning of said Act in each year of insurance coverage.

The insurance applies to the damage that becomes apparent during the study or within 4 years after the end of the study.

The insurance policies of the participating clinical sites will be valid for this study.

## Incentives

There will be no incentives for participation. Extra travel expenses due to participation will be reimbursed.

# 9. Administrative aspects and publication

## 9.1. Handling and storage of data and documents

The CRF will contain all the study related information of the patient including the completed questionnaires, results of SPT and CAP FEIA and DBPCFC. The CRF will be stored at the site under the responsibility of the investigator. After the last visit the CRF will be sent to the coordinating investigator, and all data will be entered in the database. This database is kept under the responsibility of the coordinating investigator. A data manger will review the entries and will take care of back-up several times a day.

Every patient is uniquely identified by a combination of his/her centre number (Erasmus MC nr 111; Reinier de Graaf Gasthuis nr 222; University Medical Centre Groningen nr 333), and patient number. In each centre, the first patient is assigned patient number 1, and subsequent patients are assigned consecutive numbers (2, 3 etc.). The code list is kept by the local investigator.

The study documents provided to the coordinating investigator will be coded with the centre/patient number. Information directly referring to the patient’s identity will not be supplied.

The patient’s study data will be stored for 15 years after completion of the study. The blood samples will be destroyed when the study is concluded.

## 9.2. Amendments

Amendments are changes made to the research after a favourable opinion by the accredited METC has been given. All substantial amendments will be notified to the EC that gave a favourable opinion.

Non-substantial amendments will not be notified to the accredited METC and the competent authority, but will be recorded and filed by the sponsor.

## 9.3. Annual progress report

The coordinating investigator will submit a summary of the progress of the trial to the accredited EC once a year. Information will be provided on the date of inclusion of the first subject, numbers of subjects included and numbers of subjects that have completed the trial, serious adverse events/ serious adverse reactions, other problems, and amendments.

## 9.4. End of study report

The investigator will notify the accredited EC of the end of the study within a period of 8 weeks. The end of the study is defined as the last patient’s last visit. In case the study is ended prematurely, the investigator will notify the accredited EC, including the reasons for the premature termination.

Within one year after the end of the study, the coordinating investigator will submit a final study report with the results of the study, including any publications/abstracts of the study, to the accredited EC.

## 9.5. Public disclosure and publication policy

An outline of the study protocol and a summary of the study results will be placed on the publically accessible website of the Dutch Trial Register ([www.trialregister.nl](http://www.trialregister.nl/)).

The publication policy of the CCMO (March 2002, [www.ccmo.nl](http://www.ccmo.nl/)) will be adhered to in full.

# 10. References

43. Peduzzi P, Concato J, Kemper E, Holford TR, Feinstein AR. A simulation study of the number of events per variable in logistic regression analysis. J Clin Epidemiol 1996;49:1373-9.

44. Harrell FE Jr, Lee KL, Mark DB (1996) Multivariable prognostic models: issues in developing models, evaluating assumptions and adequacy, and measuring and reducing errors. Stat Med 15:361–387.

# Attachment 1: Flow Chart

# Attachment 2: Skin Prick test result form

**SPT formulier**

| Patient ID: ___ ____  (expl. 111 0001) |
| --- |

| Initialen: ___ ___  (voorbeeld: NJ) |
| --- |

| Geslacht ⁪ |
| --- |

| Positive control | Mango sap |  |  |
| --- | --- | --- | --- |
| Positive Control | Pistache nut |  |  |
| Negative control | Peanut |  |  |
| Birch pollen |  |  |  |

#

# Attachment 3: Preparation of the DBPCFC day

**Voorbereiding van de Dubbelblinde placebogecontroleerde voedselprovocatie met cashew noot (in te vullen door verpleegkundigen)**

**2^e^ en 3^e^ lijn**

**Patiëntgegevens:**

Naam: …………………………..

Patiëntnummer: ………………………….

Geboortedatum: …………………………..

**Naam uitvoerende:** ………………………….

**Verantwoordelijke arts:**

Naam: Seinnummer:……………………….

**Arts-Achterwacht**

Naam;…………………………………… Seinnummer; ……………………

**Anamnese**

Wordt cashew noot nog steeds vermeden?

1. ja

0 nee (Indien nee: overleg met arts over indicatie van provocatie)

**Medicatie**

Medicatie gestaakt 0 ja, te weten ……………………………….

0 nee

Medicatie door gebruikt 0 ja, te weten……………………………….

1. nee

NB: Indien antihistaminica de laatste 72 uur of lokale corticosteroïden de laatste 24 uur zijn door gebruikt kan de provocatie NIET doorgaan!

**Gebruikte voeding**

Het kind heeft de laatste 3 uur niet meer gegeten of gedronken:

1. ja
2. nee, namelijk ……………………………………………………………

(zo nee, overleg met de arts)

**Allergieonderzoek :**

1. niet verricht
2. wel verricht : 0 Specifiek IgE koemelk: …….. kU/l datum:……….

0 Huidtest met koemelk ….…. mm datum ………

0 huidtest niet verricht

**Infuus**

Dient de patiënt voor de provocatie een infuus te krijgen?

0 Ja

0 nee

Reden voor infuus:

**Noodmedicatie**

Lengte:

Gewicht:

Bloeddruk:

Hartfrequentie:

*Adrenaline* (Ampul 1mg =1ml)

0,01 mg/kg intramusculair, max. 0,5 mg

………. mg = ………. ml i.m. (dijbeen anterior-lateraal)

*Clemastine® (Tavegil) )*

0,025 mg/kg intraveneus of intramusculair (max. 2 mg)

………. mg i.v**.**

*Fenistildruppels (1mg/ml)*

1–3 jaar: 15 druppels p.o.

*Xyzal (*tablet 5 mg, drank 0,5 mg/ml)

2-6 jaar: 1,25 mg = 2,5 ml

*Aerius* (tablet 5 mg, stroop 0,5 mg/ml)

1-5 jaar: 1,25 mg = 2,5 ml

Dexamethason 0,1 mg/kg (max. 8 mg) intraveneus of intramusculair

………. mg i.v./i.m.

*Hydrocortison* 4 mg/kg (max. 100 mg) intraveneus of intramusculair

………. mg i.v. /i.m.

*Salbutamol® (Ventolin) zie 1^e^ lijn dosisaerosol met voorzetkamer* (nebule 2,5 mg/2,5 ml of 5 mg/2,5 ml)

en

*Atrovent* (nebule van 500 mcg/2ml) dosisaerosol 20 ug (op indicatie aantal pufs ophogen)

via Sidestream vernevelaar met 8 liter zuurstof

<4 jaar: Ventolin 2,5 mg (nebule 2,5 mg/2,5 ml)

Atrovent 250 mcg (1 ml van nebule 500 mcg/ml**)**

>4 jaar Ventolin 5 mg (nebule 5 mg/2,5 ml)

Atrovent 500 mcg (2 ml van nebule 500 mcg/ml)

**Beoordeling conditie van de patiënt bij aanvang van de provocatie**

Naam arts/verpleegkundige: ………………………………………………

Bij aanvang van de provocatie moet het kind in een goede conditie zijn.

Afgelopen 2 weken klachten gehad? (indien ja, specificeren)

Neus/ogen 0 nee 0 ja (verbeterd, wisselend, verslechterd)

Longen 0 nee 0 ja (verbeterd, wisselend, verslechterd)

Huid 0 nee 0 ja (verbeterd, wisselend, verslechterd)

Maagdarmkanaal 0 nee 0 ja (verbeterd, wisselend, verslechterd)

Andere 0 nee 0 ja (verbeterd, wisselend, verslechterd)

Huidige medicatie naam dosis laatste gebruik

Antihistaminica ………………………………………………………..

Longmedicatie: ………………………………………………………..

ICS ………………………………………………………..

Luchtwegverwijder ………………………………………………………..

Overig ………………………………………………………..

Huid ………………………………………………………..

Vette zalf ………………………………………………………..

Steroïdcrème ………………………………………………………..

Overig ……………………………………………………….

**Lichamelijk onderzoek voor provocatie**

Algemene indruk (verkoudheden, griep, vaccinatie gehad, overig):

Hals/Hoofd:

Longen:

Huid: (*vul TIS score in*)

Temperatuur ……..°C

Piekstroom ………...l/min

Saturatie (O2) ……..%

Overig:


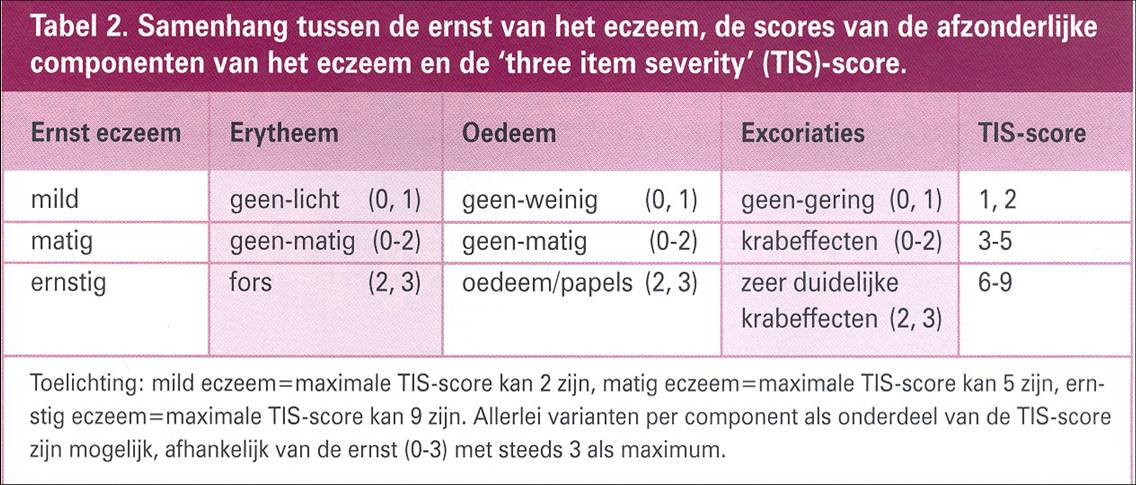


Totaal TIS score:

**Kan de provocatie vandaag doorgaan?**

0 ja 0 nee, want ……………………………………………………..

**Te ondernemen acties:**

1. IC gewaarschuwd
2. Noteren in patiëntenstatus dat provocatie vandaag plaatsvindt

0 Informed consent innemen en in status opbergen

# Attachment 4: Dosing schedule food challenge

**Doseer schema voedselprovocatie cashew in kruidkoek**

**15 gram cashew (21,2% eiwit)**

Uitgangspunten:

Doseerschema in eiwit equivalenten

Cashewnoot bevat 21,2% eiwit, dus 100 g cashew levert 21,2 g =21.200 mg eiwit.

Recept met 15 gram cashewnoot bevat dus 21,2/100*15 = 3,18 g eiwit = 3180 mg

Totale recept weegt tussen ca. 120 en 147 g (deel van het vocht verdampt).

1 cashewnoot weegt ca. 700 mg.

Totale recept (**ca. 120 g**) bevat 15 gram cashewnoot (ca. 22 cashewnoten) met **3180** mg eiwit (≥ 4 jaar)

Halve recept (ca. 60 g) bevat ca. 7,5 gram cashewnoot (ca. 11 cashewnoten) met **1590** mg eiwit (1-4 jaar)

Hierbij is uitgegaan van ca.

| Dosis | Eiwit  (mg) | Eiwit  Cum mg | Omrekening | Hoeveelheid  Recept (g) | Hoeveelheid recept  (Cum g) |
| --- | --- | --- | --- | --- | --- |
| 1 | 1 | 1 | (120*/3180) x 1 | 0,04 | 0,04 |
| 2 | 3 | 4 | (120*/3180) x 3 | 0,11 | 0,15 |
| 3 | 10 | 14 | (120*/3180) x 10 | 0,38 | 0,53 |
| 4 | 30 | 44 | (120*/3180) x 30 | 1,13 | 1,66 |
| 5 | 100 | 144 | (120*/3180) x 100 | 3,77 | 5,43 |
| 6 | 300 | 444 | (120*/3180) x 300 | 11,32 | 16,75 |
| 7 | 1000 | 1444 | (120*/3180) x 1000 | 37,74 | 1 – 4 jaar  54,49 |
| 8 | 1736 | 3180 | (120*/3180) x 1736 | restant  65,51 | ≥ 4 jaar  120,00 |

* NB: Dit moet elke keer opnieuw worden berekend, bij elke provocatie, afhankelijk van het totaal gewicht van de kruidkoek, uitgaande van 15 g cashewnoot met 3180 mg eiwit in totaal recept.

Als het gewicht van de kruidkoek afwijkt van 120 gram: dan zelf de porties uitrekenen als volgt:

Voor 1^e^ dosis: totale gewicht/3180 x 1 = …… gram recept

Voor 2^e^ dosis: totale gewicht/3180 x 3 = …… gram recept

Voor 3^e^ dosis: totale gewicht/3180 x 10 = ……. gram recept

Voor 4^e^ dosis: totale gewicht/3180 x 30 = …… gram recept

Voor 5e dosis: totale gewicht/3180 x 100 = …… gram recept

Voor 6e dosis: totale gewicht/3180 x 300 = …… gram recept

Voor 7e dosis: totale gewicht/3180 x 1000 = ……..gram recept

Voor 8e dosis: totale gewicht/3180 x 1736 = …… gram recept

# Attachment 5: Scoring system DBPCFC

**symptoom registratie formulier dubbelblinde placebogecontroleerde provocatie MET CASHEW NOOT**

**IDEAL STUDIE - TEST DAG 1**

| Patient ID: ___ ____  (expl. 111 0001) |
| --- |

| Initialen: ___ ___  (voorbeeld: NJ) |
| --- |

| Geslacht ⁪ |
| --- |

**Testdatum:………………………………………………….**

**0 Type testvoeding : …………………………………**

**0 Dubbelblinde test, testdag 1**

**0 Infuus hoeft niet ingebracht**

**0 Infuus ingebracht**

Voorbeeld registratie van symptomen:

ernst score 1 = mild

2 = matig

3 = ernstig

|  | **Dosis 1**  **Tijd:** | **Reactie**  **Tijd:** | **Dosis 2**  **Tijd:** | **Reactie**  **Tijd:** | **Dosis 3**  **Tijd:** | **Reactie**  **Tijd:** | **Dosis 4**  **Tijd:** | **Reactie**  **Tijd:** | **Dosis 5**  **Tijd:** | **Reactie**  **Tijd:** | **Dosis 6**  **Tijd:** | **Reactie**  **Tijd:** | **Dosis 7**  **Tijd:** | **Reactie**  **Tijd:** | **Dosis 8**  **Tijd:** | **Reactie**  **Tijd:** |
| --- | --- | --- | --- | --- | --- | --- | --- | --- | --- | --- | --- | --- | --- | --- | --- | --- |
| **Tijdstip** | **9.00** |  | **9.30** |  | **10.00** |  |  |  |  |  |  |  |  |  |  |  |
| **Symptomen** |  |  |  |  |  |  |  |  |  |  |  |  |  |  |  |  |
| Jeuk |  |  |  |  |  | **10.20**  **2** |  |  |  |  |  |  |  |  |  |  |

|  | **Dosis 1**  **Tijd:** | **Reactie**  **Tijd:** | **Dosis 2**  **Tijd:** | **Reactie**  **Tijd:** | **Dosis 3**  **Tijd:** | **Reactie**  **Tijd:** | **Dosis 4**  **Tijd:** | **Reactie**  **Tijd:** | **Dosis 5**  **Tijd:** | **Reactie**  **Tijd:** | **Dosis 6**  **Tijd:** | **Reactie**  **Tijd:** | **Dosis 7**  **Tijd:** | **Reactie**  **Tijd:** | **Dosis 8**  **Tijd:** | **Reactie**  **Tijd:** |
| --- | --- | --- | --- | --- | --- | --- | --- | --- | --- | --- | --- | --- | --- | --- | --- | --- |
| **Tijdstip** |  |  |  |  |  |  |  |  |  |  |  |  |  |  |  |  |
| **Symptomen** |  |  |  |  |  |  |  |  |  |  |  |  |  |  |  |  |
| Jeuk |  |  |  |  |  |  |  |  |  |  |  |  |  |  |  |  |
| Roodheid |  |  |  |  |  |  |  |  |  |  |  |  |  |  |  |  |
| Lokale  urticaria |  |  |  |  |  |  |  |  |  |  |  |  |  |  |  |  |
| Gegenerali-  seerde urticaria |  |  |  |  |  |  |  |  |  |  |  |  |  |  |  |  |
| Zwelling |  |  |  |  |  |  |  |  |  |  |  |  |  |  |  |  |
| Overig |  |  |  |  |  |  |  |  |  |  |  |  |  |  |  |  |
| **Gastro-intestinaal** |  |  |  |  |  |  |  |  |  |  |  |  |  |  |  |  |
| Angio-oedeem van de lippen, tong en gehemelte |  |  |  |  |  |  |  |  |  |  |  |  |  |  |  |  |
| Jeuk in de mond |  |  |  |  |  |  |  |  |  |  |  |  |  |  |  |  |
| (Krampende) buikpijn |  |  |  |  |  |  |  |  |  |  |  |  |  |  |  |  |
| Misselijkheid |  |  |  |  |  |  |  |  |  |  |  |  |  |  |  |  |
| Spugen (eenmalig) |  |  |  |  |  |  |  |  |  |  |  |  |  |  |  |  |
|  | **Dosis 1**  **Tijd:** | **Reactie**  **Tijd:** | **Dosis 2**  **Tijd:** | **Reactie**  **Tijd:** | **Dosis 3**  **Tijd:** | **Reactie**  **Tijd:** | **Dosis 4**  **Tijd:** | **Reactie**  **Tijd:** | **Dosis 5**  **Tijd:** | **Reactie**  **Tijd:** | **Dosis 6**  **Tijd:** | **Reactie**  **Tijd:** | **Dosis 7**  **Tijd:** | **Reactie**  **Tijd:** | **Dosis 8**  **Tijd:** | **Reactie**  **Tijd:** |
| Herhaald spugen |  |  |  |  |  |  |  |  |  |  |  |  |  |  |  |  |
| Diarree |  |  |  |  |  |  |  |  |  |  |  |  |  |  |  |  |
| Overig |  |  |  |  |  |  |  |  |  |  |  |  |  |  |  |  |
| **Respiratoir** |  |  |  |  |  |  |  |  |  |  |  |  |  |  |  |  |
| Neusobstructie |  |  |  |  |  |  |  |  |  |  |  |  |  |  |  |  |
| Niezen |  |  |  |  |  |  |  |  |  |  |  |  |  |  |  |  |
| Conjunctivitis |  |  |  |  |  |  |  |  |  |  |  |  |  |  |  |  |
| Loopneus |  |  |  |  |  |  |  |  |  |  |  |  |  |  |  |  |
| Gevoel van zwelling keel |  |  |  |  |  |  |  |  |  |  |  |  |  |  |  |  |
| Stridor/ Heesheid |  |  |  |  |  |  |  |  |  |  |  |  |  |  |  |  |
| Hoesten |  |  |  |  |  |  |  |  |  |  |  |  |  |  |  |  |
| Moeilijk slikken |  |  |  |  |  |  |  |  |  |  |  |  |  |  |  |  |
| Benauwdheid |  |  |  |  |  |  |  |  |  |  |  |  |  |  |  |  |
| Astma aanval |  |  |  |  |  |  |  |  |  |  |  |  |  |  |  |  |
| Overige |  |  |  |  |  |  |  |  |  |  |  |  |  |  |  |  |
| **Cardio-**  **Vasculair** |  |  |  |  |  |  |  |  |  |  |  |  |  |  |  |  |
| Tachycardie |  |  |  |  |  |  |  |  |  |  |  |  |  |  |  |  |
|  | **Dosis 1**  **Tijd:** | **Reactie**  **Tijd:** | **Dosis 2**  **Tijd:** | **Reactie**  **Tijd:** | **Dosis 3**  **Tijd:** | **Reactie**  **Tijd:** | **Dosis 4**  **Tijd:** | **Reactie**  **Tijd:** | **Dosis 5**  **Tijd:** | **Reactie**  **Tijd:** | **Dosis 6**  **Tijd:** | **Reactie**  **Tijd:** | **Dosis 7**  **Tijd:** | **Reactie**  **Tijd:** | **Dosis 8**  **Tijd:** | **Reactie**  **Tijd:** |
| Slap worden |  |  |  |  |  |  |  |  |  |  |  |  |  |  |  |  |
| Hypotensie |  |  |  |  |  |  |  |  |  |  |  |  |  |  |  |  |
| Verminderd  bewustzijn |  |  |  |  |  |  |  |  |  |  |  |  |  |  |  |  |
| Acute gedrags-  verandering |  |  |  |  |  |  |  |  |  |  |  |  |  |  |  |  |
| overig |  |  |  |  |  |  |  |  |  |  |  |  |  |  |  |  |

**Dosis herhaald?**

**0 nee 0 ja, namelijk dosis: …………….**

**tijdstip: ………………………….**

**Laatst gegeven testvoeding:**

Volledig gegeten: 0 ja 0 nee

Indien nee: hoeveel testvoeding gegeten : ………………g

Hoeveel testvoeding over: ……………………. g

Toelichting:

……………………………………………………………………………………………

……………………………………………………………………………………………

……………………………………………………………………………………………

……………………………………………………………………………………………

……………………………………………………………………………………………

**Samenvatting reacties op de testdag**

0 geen verschijnselen

0 Verschijnselen optredend na dosis nr.: …

Medicatie gegeven? 0 nee, 0 ja, namelijk 0 antihistaminicum per os/iv

0 luchtwegverwijders

0 adrenaline

0 anders, nl.: …………………….

**Lichamelijk onderzoek aan einde testdag**

Naam beoordelaar:

Algemene indruk:

Hals/Hoofd:

Longen:

Huid: (*vul TIS score in*)

Bloeddruk:

Hartfrequentie:

Temperatuur ……..°C

Piekstroom ………...l/min

Saturatie (O2) ……..%

Overig:

**Te ondernemen acties:**

0 Belafspraak met ouders voor over 48 uur

0 Symptoomscore formulier voor thuis meegeven

0 Check bij de ouders of ze weten wat ze moeten doen bij reacties thuis (contact gegevens – zie Patiënteninformatie)

0 Check bij de ouders dat ze weten dat ze het cashew noot nog moeten vermijden

0 Voorlopige uitslag provocatie in status noteren

**(Telefonische) Controle na 48 uur**

Datum:

Naam arts/verpleegkundige:

Sinds provocatie klachten veranderd? Indien ja, specificeren.

Neus/ogen 0 nee 0 ja, namelijk ……………………………………………………………………………….

Longen 0 nee 0 ja, namelijk ……………………………………………………………………………….

Huid 0 nee 0 ja, namelijk ……………………………………………………………………………….

Andere 0 nee 0 ja, namelijk ……………………………………………………………………………….

Wanneer zijn deze klachten opgetreden (hoeveel uren na provocatie)?

Medicatie veranderd sinds laatste provocatie? Indien ja, specificeren.

**Objectivatie symptomen (bij optredende symptomen binnen 48 uur)**

Datum:

Tijdstip:

Naam arts/verpleegkundige:

Neus/ogen 0 nee 0 ja, te weten ………………………………

Longen 0 nee 0 ja, te weten ………………………………

Huid 0 nee 0 ja, te weten ………………………………

Andere 0 nee 0 ja te weten……………………………….

0 Symptoomscore formulier voor thuis opgeborgen in het dossier van de patiënt.

**Conclusie provocatie dag**

Provocatie is reglementair afgerond ( tenminste 75% van de laatst gegeven dosis gegeten of gedronken): 0 ja 0 nee

Indien ja:

1. Er is een objectieve reactie opgetreden
2. Er zijn herhaalde of persisterende subjectieve reactie opgetreden
3. Er zijn geen reacties opgetreden

Indien nee:

0 het kind weigerde de voeding volledig te eten

0 De provocatie is gestaakt op verzoek op de ouders

0 Anders, te weten ……………………………….

**Beoordeling provocatie dag:**

1. De provocatiedag is positief
2. De provocatie dag is negatief
3. De provocatie dag is dubieus of onbeslist

Toelichting:

Een provocatie is geslaagd als tenminste t/m 75% van de laatste provocatiedosis is ingenomen.

Een provocatie is negatief of onbeslist bij milde objectieve of aanhoudende subjectieve klachten op de laatste provocatie dosis.

**symptoom registratie formulier dubbelblinde placebogecontroleerde provocatie MET CASHEW NOOT**

**IDEAL STUDIE - TEST DAG 2**

**Naam kind: …………………………………………………**

**Patiënt nummer**

**Geboorte datum: ………………………………………….**

**Testdatum:………………………………………………….**

**0 Type testvoeding : …………………………………**

**0 Dubbelblinde test, testdag 1**

**0 Infuus hoeft niet ingebracht**

**0 Infuus ingebracht**

Voorbeeld registratie van symptomen:

ernst score 1 = mild

2 = matig

3 = ernstig

|  | **Dosis 1**  **Tijd:** | **Reactie**  **Tijd:** | **Dosis 2**  **Tijd:** | **Reactie**  **Tijd:** | **Dosis 3**  **Tijd:** | **Reactie**  **Tijd:** | **Dosis 4**  **Tijd:** | **Reactie**  **Tijd:** | **Dosis 5**  **Tijd:** | **Reactie**  **Tijd:** | **Dosis 6**  **Tijd:** | **Reactie**  **Tijd:** | **Dosis 7**  **Tijd:** | **Reactie**  **Tijd:** | **Dosis 8**  **Tijd:** | **Reactie**  **Tijd:** |
| --- | --- | --- | --- | --- | --- | --- | --- | --- | --- | --- | --- | --- | --- | --- | --- | --- |
| **Tijdstip** | **9.00** |  | **9.30** |  | **10.00** |  |  |  |  |  |  |  |  |  |  |  |
| **Symptomen** |  |  |  |  |  |  |  |  |  |  |  |  |  |  |  |  |
| Jeuk |  |  |  |  |  | **10.20**  **2** |  |  |  |  |  |  |  |  |  |  |

|  | **Dosis 1**  **Tijd:** | **Reactie**  **Tijd:** | **Dosis 2**  **Tijd:** | **Reactie**  **Tijd:** | **Dosis 3**  **Tijd:** | **Reactie**  **Tijd:** | **Dosis 4**  **Tijd:** | **Reactie**  **Tijd:** | **Dosis 5**  **Tijd:** | **Reactie**  **Tijd:** | **Dosis 6**  **Tijd:** | **Reactie**  **Tijd:** | **Dosis 7**  **Tijd:** | **Reactie**  **Tijd:** | **Dosis 8**  **Tijd:** | **Reactie**  **Tijd:** |
| --- | --- | --- | --- | --- | --- | --- | --- | --- | --- | --- | --- | --- | --- | --- | --- | --- |
| **Tijdstip** |  |  |  |  |  |  |  |  |  |  |  |  |  |  |  |  |
| **Symptomen** |  |  |  |  |  |  |  |  |  |  |  |  |  |  |  |  |
| Jeuk |  |  |  |  |  |  |  |  |  |  |  |  |  |  |  |  |
| Roodheid |  |  |  |  |  |  |  |  |  |  |  |  |  |  |  |  |
| Lokale  urticaria |  |  |  |  |  |  |  |  |  |  |  |  |  |  |  |  |
| Gegenerali-  seerde urticaria |  |  |  |  |  |  |  |  |  |  |  |  |  |  |  |  |
| Zwelling |  |  |  |  |  |  |  |  |  |  |  |  |  |  |  |  |
| Overig |  |  |  |  |  |  |  |  |  |  |  |  |  |  |  |  |
| **Gastro-intestinaal** |  |  |  |  |  |  |  |  |  |  |  |  |  |  |  |  |
| Angio-oedeem van de lippen, tong en gehemelte |  |  |  |  |  |  |  |  |  |  |  |  |  |  |  |  |
| Jeuk in de mond |  |  |  |  |  |  |  |  |  |  |  |  |  |  |  |  |
| (Krampende) buikpijn |  |  |  |  |  |  |  |  |  |  |  |  |  |  |  |  |
| Misselijkheid |  |  |  |  |  |  |  |  |  |  |  |  |  |  |  |  |
| Spugen (eenmalig) |  |  |  |  |  |  |  |  |  |  |  |  |  |  |  |  |
|  | **Dosis 1**  **Tijd:** | **Reactie**  **Tijd:** | **Dosis 2**  **Tijd:** | **Reactie**  **Tijd:** | **Dosis 3**  **Tijd:** | **Reactie**  **Tijd:** | **Dosis 4**  **Tijd:** | **Reactie**  **Tijd:** | **Dosis 5**  **Tijd:** | **Reactie**  **Tijd:** | **Dosis 6**  **Tijd:** | **Reactie**  **Tijd:** | **Dosis 7**  **Tijd:** | **Reactie**  **Tijd:** | **Dosis 8**  **Tijd:** | **Reactie**  **Tijd:** |
| Herhaald spugen |  |  |  |  |  |  |  |  |  |  |  |  |  |  |  |  |
| Diarree |  |  |  |  |  |  |  |  |  |  |  |  |  |  |  |  |
| Overig |  |  |  |  |  |  |  |  |  |  |  |  |  |  |  |  |
| **Respiratoir** |  |  |  |  |  |  |  |  |  |  |  |  |  |  |  |  |
| Neusobstructie |  |  |  |  |  |  |  |  |  |  |  |  |  |  |  |  |
| Niezen |  |  |  |  |  |  |  |  |  |  |  |  |  |  |  |  |
| Conjunctivitis |  |  |  |  |  |  |  |  |  |  |  |  |  |  |  |  |
| Loopneus |  |  |  |  |  |  |  |  |  |  |  |  |  |  |  |  |
| Gevoel van zwelling keel |  |  |  |  |  |  |  |  |  |  |  |  |  |  |  |  |
| Stridor/ Heesheid |  |  |  |  |  |  |  |  |  |  |  |  |  |  |  |  |
| Hoesten |  |  |  |  |  |  |  |  |  |  |  |  |  |  |  |  |
| Moeilijk slikken |  |  |  |  |  |  |  |  |  |  |  |  |  |  |  |  |
| Benauwdheid |  |  |  |  |  |  |  |  |  |  |  |  |  |  |  |  |
| Astma aanval |  |  |  |  |  |  |  |  |  |  |  |  |  |  |  |  |
| Overige |  |  |  |  |  |  |  |  |  |  |  |  |  |  |  |  |
| **Cardio-**  **Vasculair** |  |  |  |  |  |  |  |  |  |  |  |  |  |  |  |  |
| Tachycardie |  |  |  |  |  |  |  |  |  |  |  |  |  |  |  |  |
|  | **Dosis 1**  **Tijd:** | **Reactie**  **Tijd:** | **Dosis 2**  **Tijd:** | **Reactie**  **Tijd:** | **Dosis 3**  **Tijd:** | **Reactie**  **Tijd:** | **Dosis 4**  **Tijd:** | **Reactie**  **Tijd:** | **Dosis 5**  **Tijd:** | **Reactie**  **Tijd:** | **Dosis 6**  **Tijd:** | **Reactie**  **Tijd:** | **Dosis 7**  **Tijd:** | **Reactie**  **Tijd:** | **Dosis 8**  **Tijd:** | **Reactie**  **Tijd:** |
| Slap worden |  |  |  |  |  |  |  |  |  |  |  |  |  |  |  |  |
| Hypotensie |  |  |  |  |  |  |  |  |  |  |  |  |  |  |  |  |
| Verminderd  bewustzijn |  |  |  |  |  |  |  |  |  |  |  |  |  |  |  |  |
| Acute gedrags-  verandering |  |  |  |  |  |  |  |  |  |  |  |  |  |  |  |  |
| overig |  |  |  |  |  |  |  |  |  |  |  |  |  |  |  |  |

**Dosis herhaald?**

**0 nee 0 ja, namelijk dosis: …………….**

**tijdstip: ………………………….**

**Laatst gegeven testvoeding:**

Volledig gegeten: 0 ja 0 nee

Indien nee: hoeveel testvoeding gegeten : ………………g

Hoeveel testvoeding over: ……………………. g

Toelichting:

……………………………………………………………………………………………

……………………………………………………………………………………………

……………………………………………………………………………………………

……………………………………………………………………………………………

……………………………………………………………………………………………

**Samenvatting reacties op de testdag**

0 geen verschijnselen

0 Verschijnselen optredend na dosis nr.: …

Medicatie gegeven? 0 nee, 0 ja, namelijk 0 antihistaminicum per os/iv

0 luchtwegverwijders

0 adrenaline

0 anders, nl.: …………………….

**Lichamelijk onderzoek aan einde testdag**

Naam beoordelaar:

Algemene indruk:

Hals/Hoofd:

Longen:

Huid: (*vul TIS score in*)

Bloeddruk:

Hartfrequentie:

Temperatuur ……..°C

Piekstroom ………...l/min

Saturatie (O2) ……..%

Overig:

**Te ondernemen acties:**

0 Belafspraak met ouders voor over 48 uur

0 Symptoomscore formulier voor thuis meegeven

0 Check bij de ouders of ze weten wat ze moeten doen bij reacties thuis (contact gegevens – zie Patiënteninformatie)

0 Check bij de ouders dat ze weten dat ze het cashew noot nog moeten vermijden

0 Voorlopige uitslag provocatie in status noteren

**(Telefonische) Controle na 48 uur**

Datum:

Naam arts/verpleegkundige:

Sinds provocatie klachten veranderd? Indien ja, specificeren.

Neus/ogen 0 nee 0 ja, namelijk ……………………………………………………………………………….

Longen 0 nee 0 ja, namelijk ……………………………………………………………………………….

Huid 0 nee 0 ja, namelijk ……………………………………………………………………………….

Andere 0 nee 0 ja, namelijk ……………………………………………………………………………….

Wanneer zijn deze klachten opgetreden (hoeveel uren na provocatie)?

Medicatie veranderd sinds laatste provocatie? Indien ja, specificeren.

**Objectivatie symptomen (bij optredende symptomen binnen 48 uur)**

Datum:

Tijdstip:

Naam arts/verpleegkundige:

Neus/ogen 0 nee 0 ja, te weten ………………………………

Longen 0 nee 0 ja, te weten ………………………………

Huid 0 nee 0 ja, te weten ………………………………

Andere 0 nee 0 ja te weten……………………………….

0 Symptoomscore formulier voor thuis opgeborgen in het dossier van de patiënt.

**Conclusie provocatie dag**

Provocatie is reglementair afgerond ( tenminste 75% van de laatst gegeven dosis gegeten of gedronken): 0 ja 0 nee

Indien ja:

1. Er is een objectieve reactie opgetreden
2. Er zijn herhaalde of persisterende subjectieve reactie opgetreden
3. Er zijn geen reacties opgetreden

Indien nee:

0 het kind weigerde de voeding volledig te eten

0 De provocatie is gestaakt op verzoek op de ouders

0 Anders, te weten ……………………………….

**Beoordeling provocatie dag:**

1. De provocatiedag is positief
2. De provocatie dag is negatief
3. De provocatie dag is dubieus of onbeslist

Toelichting:

Een provocatie is geslaagd als tenminste t/m 75% van de laatste provocatiedosis is ingenomen.

Een provocatie is negatief of onbeslist bij milde objectieve of aanhoudende subjectieve klachten op de laatste provocatie dosis.

**EINDBEOORDELING DUBBELBLINDE PLACEBOGECONTROLEERDE PROVOCATIE MET CASHEWNOOT**

0 Verbreek de code: 0 Provocatie dag 1 was verum / placebo

0 Provocatie dag 2 was verum / placebo

**Eindbeoordeling provocatie test**

0 Eindbeoordeling DBPGVP: ……………………………………….(positief, negatief of onbeslist)

**Toelichting**

| **Verum** | **Placebo** | **Eindbeoordeling DBPGVP** |
| --- | --- | --- |
| Positief | Negatief | Positief |
| Negatief | Negatief | Negatief |
| Negatief (of positief, maar minder positief dan placebo) | Positief | Negatief |
| Positief | Positief | Negatief of onbeslist* |

* Overleg met een gespecialiseerd centrum: Bij duidelijke verum reacties is de uitslag onbeslist en wordt de DBPCFC herhaald.

* Bij minder duidelijke of vertraagde verum reacties is de uitslag negatief of onbeslist.

Een provocatie is geslaagd als tenminste t/m 75% van de laatste provocatiedosis is ingenomen.

**FOLLOW-UP** **DUBBELBLINDE PLACEBOGECONTROLEERDE PROVOCATIE MET CASHEWNOOT**

**Vervolg advies en te ondernemen acties: Acties**

1. Cashewnootvrij dieet handhaven (bij positieve test) Verwijs zo nodig naar de diëtiste
2. Cashewnoot in dieet introduceren (bij negatieve test) Advies thuis introductie Cashew
3. Informeer de huisarts, consultatiebureau arts, kinderarts en diëtiste Schrijf rapportage

# Attachment 6: Symptom score 48 h post challenge

**Symptoom score formulier voor thuis (Voor 48 uur na de provocatie)**

**IDEAL studie**

| Patient ID: ___ ____  (expl. 111 0001) |
| --- |

| Initialen: ___ ___  (voorbeeld: NJ) |
| --- |

| Geslacht ⁪ |
| --- |

**Na de voedselprovocatie:**

48 uur na afloop van de test vragen wij u contact met ons op te nemen om te bespreken hoe het thuis is gegaan. Hiervoor maken wij een telefonische afspraak.

Wij vragen u om eventueel optredende klachten te noteren op het klachten registratie formulier (zie hieronder) dat u na de provocatie mee krijgt.

Als uw kind binnen 48 uur na de provocatie duidelijke klachten krijgt vragen wij u contact op te nemen met de arts- onderzoeker:

……………………………………………………………………, telefoon

Na de voedselprovocatie houdt u zich nog strikt aan het dieet. Na afloop van de hele provocatietest wordt de uitslag met u besproken. Afhankelijk van de test uitslag wordt hierna het cashewnoot vrije dieet wordt voortgezet, of dat cashewnoot weer wordt geïntroduceerd in het dieet. Hiervoor komt u nog 1 x naar de kliniek. U ontvangt hiervoor nadere mondelinge en schriftelijke richtlijnen.

Bij twijfel, eventuele reacties thuis of vragen kunt u altijd eerder contact opnemen met het de arts/verpleegkundigen op tel nr … of bel de huisarts.

Noteer hieronder zo nauwkeurig mogelijk welke verschijnselen er gedurende de 48 uur (2 dagen) na de provocatie op traden, en of u medicatie heeft gegeten:

| Datum | Tijdstip | Verschijnselen/allergische klachten: | Medicatie gegeven?  Naam, dosering, tijdstip |
| --- | --- | --- | --- |
|  |  |  |  |
